# Characterization of a Novel Monoclonal Antibody for Serine-129 Phosphorylated α-Synuclein: A Potential Application for Clinical and Basic Research

**DOI:** 10.3389/fneur.2022.821792

**Published:** 2022-02-17

**Authors:** Weijin Liu, Qidi Zhang, Hao Xing, Ge Gao, Jia Liu, Yue Huang, Hui Yang

**Affiliations:** ^1^Department of Neurobiology, School of Basic Medical Sciences, Capital Medical University, Beijing Key Laboratory of Neural Regeneration and Repair, Beijing Key Laboratory on Parkinson's Disease, Key Laboratory for Neurodegenerative Disease of the Ministry of Education, Beijing Institute of Brain Disorders, Collaborative Innovation Center for Brain Disorders, Beijing, China; ^2^Laboratory of Brain Disorders, Ministry of Science and Technology, Collaborative Innovation Center for Brain Disorders, Beijing Institute of Brain Disorders, Capital Medical University, Beijing, China; ^3^China National Clinical Research Center for Neurological Diseases, Beijing Tiantan Hospital, Beijing, China

**Keywords:** α-synuclein, Lewy bodies, Parkinson's disease, phosphorylation, phosphospecific antibody

## Abstract

The Lewy bodies (LBs) are the pathological hallmark of Parkinson's disease (PD). More than 90% of α-synuclein (α-syn) within LBs is phosphorylated at the serine-129 residue [pSer129 α-syn (p-α-syn)]. Although various studies have revealed that this abnormally elevated p-α-syn acts as a pathological biomarker and is involved in the pathogenic process of PD, the exact pathophysiological mechanisms of p-α-syn are still not fully understood. Therefore, the development of specific and reliable tools for p-α-syn detection is important. In this study, we generated a novel p-α-syn mouse monoclonal antibody (C140S) using hybridoma technology. To further identify the characteristics of C140S, we performed several *in vitro* assays using recombinant proteins, along with *ex vivo* assays utilizing the brains of Thy1-SNCA transgenic (Tg) mice, the preformed fibril (PFF)-treated neurons, and the brain sections of patients with PD. Our C140S specifically recognized human and mouse p-α-syn proteins both *in vitro* and *ex vivo*, and similar to commercial p-α-syn antibodies, the C140S detected higher levels of p-α-syn in the midbrain of the Tg mice. Using immunogold electron microscopy, these p-α-syn particles were partly deposited in the cytoplasm and colocalized with the outer mitochondrial membrane. In addition, the C140S recognized p-α-syn pathologies in the PFF-treated neurons and the amygdala of patients with PD. Overall, the C140S antibody was a specific and potential research tool in the detection and mechanistic studies of pathogenic p-α-syn in PD and related synucleinopathies.

## Introduction

Parkinson's disease (PD) is one of the most common movement disorders in the world. Characteristic pathological changes of PD include the formation of intracellular inclusions termed, “Lewy bodies” (LBs) and “Lewy neurites” (LNs) (1, 2). The major component of these inclusions is α-synuclein (α-syn). In normal physiological circumstances, α-syn is an “intrinsically disordered protein,” that is highly dynamic in conformation (3). Under pathological conditions, misfolded α-syn can lead to the formation of different α-syn strains, which might have damaging effects within the neurons (1, 4). Despite intense research efforts, little is known about how α-syn abnormally aggregates and finally becomes the main component of LBs and LNs in the relevant brain regions. Moreover, pathological forms of α-syn can transfer from cell-to-cell in a prion-like manner, which might further contribute to the spread of α-syn pathology and promote the neurodegenerative process (5–7). While it is not clear what mechanisms regulate α-syn transmission, we still know little about the pathophysiological roles of the secreted α-syn.

It was reported that posttranslational modifications of α-syn, especially the phosphorylation of serine-129-site (pSer129 α-syn, p-α-syn), could regulate α-syn aggregation and toxicity in disease, and p-α-syn was also involved in the pathological propagation of α-syn. Previous experiments have revealed that approximately 90% of α-syn deposited in LBs was phosphorylated at serine-129 (pSer129 α-syn, p-α-syn), whereas only 4% was modified at this residue in the soluble components of normal brains (8, 9). Currently, it is unclear why extensive phosphorylation occurs in the pathological process of PD, and the role of p-α-syn remains controversial. Although some studies suggested that p-α-syn acted as a protective agent against α-syn aggregation and neurotoxicity (10, 11), others suggested that this abnormally elevated p-α-syn was crucial in mediating α-syn neurotoxicity and inclusion formation (12, 13). In addition to the above roles, p-α-syn also functions in cell-to-cell transmission of the pathological forms of α-syn. Phosphorylation at serine-129 was reported to induce the formation of distinct α-syn strains (14), and phosphorylated exogenous α-syn fibrils could exacerbate pathology and induce neuronal dysfunction in mice (15). A recent report also concluded that p-α-syn enhanced the interaction between α-syn fibrils and its receptors, which meant that p-α-syn could facilitate the spread of α-syn pathologies (16). Overall, the role of p-α-syn is relatively complicated, and further studies are still needed to reveal its exact mechanism involved in PD pathologies.

Currently, the development of specific and reliable antibodies for p-α-syn is of high importance. An increasing number of investigators have used p-α-syn antibodies to detect α-syn pathologies in multiple peripheral tissues and biofluids of patients with PD during the preclinical phase (17–19), which suggested the potential of p-α-syn as a diagnostic or progression biomarker for PD (20, 21). A series of p-α-syn antibodies has also been used to detect pathological α-syn inclusions in PD models, which facilitated the understanding of the pathogenesis of p-α-syn (22–24). However, the exact pathophysiological mechanisms of p-α-syn are still unclear, as there are no uniform approaches and antibodies for p-α-syn detection, and some p-α-syn antibodies can cross-react with nonspecific antigens (25–27). In the present study, we generated and identified a series of mouse monoclonal p-α-syn antibodies, and detected their specificities by comparing them with commercial antibodies. Then, we used the selected p-α-syn antibody to detect α-syn pathologies in the midbrains of Thy1-SNCA transgenic (Tg) mice, and used immunogold electron microscopy to detect the ultrastructural localization of p-α-syn. Next, we used C140S to recognize p-α-syn pathologies in the preformed fibril (PFF)-treated neurons and the brain sections of patients with PD. We then highlighted the potential applications of our p-α-syn antibody in the detection and mechanistic studies of p-α-syn in the related PD pathologies.

## Materials and Methods

### Production of Monoclonal Antibodies to p-α-Syn

Generation of hybridoma cells was first performed. Briefly, human immunopeptides 1 (P1; Ac-CEAYEMP(pS)EGG-NH2; amino acids: 123–132 of p-α-syn) and peptides 2 (P2; Ac-EMP(pS)EEGYQDC-NH2; amino acids: 126–135 of p-α-syn) were emulsified with Freund's adjuvant and were repeatedly used to immunize female BALB/c mice subcutaneously. Spleen cells of mouse were selected and fused with Sp2/0 myeloma cells to generate the antibody-producing hybridomas, and 17 strains of hybridomas with satisfactory sensitivity were screened out using indirect enzyme-linked immunosorbent assays (ELISAs). We finally chose three candidate strains: C140S, C48S, and C54S for further detection. Each strain was intraperitoneally injected into female BALB/c-nu mice for antibody amplification. The collected ascites were then purified using Protein G-agarose affinity chromatography (Sigma-Aldrich; St. Louis, MO, USA).

### Cloning, Expression, and Purification of Proteins

Full-length α-syn/β-syn cDNA was cloned into the pGEX-4T-1 expression vector and transformed into BL21 (DE3) competent cells (CB105-01; Tiangen, Beijing, China). GST-α-syn/β-syn was expressed after induction with 0.1 mM isopropyl-β-D-1-thiogalactopyranoside (IPTG). The purified recombinant protein was extracted from the bacterial lysate by combining with glutathione Sepharose^TM^ 4B (GE Health Life Sciences, US). The α-syn/β-syn was collected after digestion with human thrombin for 6 h at room temperature. The elution buffer, the phosphate-buffered saline (PBS) (140 mM NaCl, 2.7 mM KCl, 10 mM Na_2_HPO_4_, 1.8 mM KH_2_PO_4_, pH 7.3) for α-syn was changed into a working buffer (20 μM HEPES, 10 μM MgCl_2_, 20 μM of dithiothreitol, pH 7.4) with a 10 kD molecular weight cut off filter (Millipore, Bedford, MA, USA). The purity of the collected α-syn was assessed with Coomassie Brilliant Blue staining and western blotting. The concentration of the α-syn/β-syn protein was tested with a bicinchoninic acid (BCA) Protein Assay Kit (Thermo Scientific Waltham, MA, USA). Aliquots of α-syn/β-syn were stored at −80°C.

### Coomassie Brilliant Blue Staining

We used Coomassie Brilliant Blue staining to assess the purity of the recombinant h-α-syn. After electrophoresis, the gels were incubated with Bio-Safe Coomassie Stain (161-0786; Bio-Rad, Hercules, CA, USA) for 1 h at room temperature. The membranes were visualized in a Gel Doc^TM^ Imager system (Bio-Rad, CA, USA).

### Preparation of p-α-Syn

To produce p-α-syn monomer, we used Polo-like-kinase 3 (PLK3), which was reported to phosphorylate α-syn at serine-129 *in vitro* (28). The reaction system consisted of 50 μL α-syn, 1.2 μL of PLK3 (Thermo Scientific, MA, USA), and 0.5 μL of adenosine triphosphate (ATP) (Sigma Aldrich, MO, USA). After incubation for 3 h in a 30°C water bath, the reaction was terminated by 25 mM ethylenediaminetetraacetic acid disodium salt (EDTA-Na_2_). The resultant p-α-syn was verified with a p-α-syn antibody using western blotting. The phosphorylation state of α-syn was further identified by mass spectrum (Supported by proteomics platform, Institute of Genetics and Development Biology, Chinese Academy of Sciences). The p-α-syn monomer was then stored at −80°C.

### Dot Blots

We used P1, P2, α-syn, p-α-syn, and β-syn to test the specificity of C140S, C48S, and C54S. Protein samples (250 and 500 ng) were spotted onto nitrocellulose membranes. The membranes were incubated with 5% nonfat dried milk (Applygen Technology, Beijing, China) on a horizontal shaker for 1 h at room temperature. The blocking solution was then removed and replaced with primary antibodies. After incubation at 4°C overnight, the membranes were washed with Tween 20 in tris-buffered saline (TBST [2% TBS; 10 mM Tris-HCl, pH 7.5, 150 mM NaCl) for three times and incubated with alkaline phosphatase (AP) conjugated secondary antibody, mouse AP-IgG (E-2636; mAP-IgG; Sigma Aldrich, MO, USA), diluted at 1:1,000. BCIP/NBT (B3804, Sigma Aldrich, USA) was used to detect AP to reflect the immunoreactivity of C140S, C48S, and C54S.

### Indirect Enzyme-Linked Immunosorbent Assays

Indirect ELISAs were conducted to verify the specificity of antibodies. A 96-well polystyrene plate (Corning, NY, USA) was coated with immunopeptides and antigens, at 100 μL/well, and incubated at 4°C overnight. Coating buffer [citrate-buffered saline (CBS), 200 mM NaHCO_3_, pH 9.6] was used to dilute protein samples. The plate was then washed three times (5 min each) with PBST (0.2% Tween-20 in 0.01 M PBS), at 200 μL/well, and then incubated with 100 μL/well of 5% bovine serum albumin (BSA) (Sigma Aldrich, MO, USA) dissolved with PBST for 2 h at 37°C. After washing, 100 μL of detection antibody was added, and the plate was incubated for 2 h at 37°C. Next, the plate was washed and incubated at 37°C for 1 h with 100 μL/well of alkaline phosphatase (AP) conjugated secondary antibody (mouse AP-IgG, E-2636/rabbit AP-IgG, SA00002-2; Sigma Aldrich, MO, USA), diluted at 1:1,000. After being washed three times, each well-received 100 μL of enzyme-substrate p-nitrophenyl phosphate (pNPP; N1891; Sigma Aldrich, MO, USA) to react with AP-IgG. The reaction was allowed to proceed for 30 min at 37°C in a dark environment, after which the absorbance was read at 405 nm with a Multiskan MK3 microplate reader (Thermo Scientific, MA, USA).

### Antibody Absorption Experiment

To test the specificity and affinity of C140S, a total of 5 μg h of p-α-syn protein was incubated with different concentrations of C140S antibody (3.2, 16, 80, 400 μg/mL) and control mouse IgG (m-IgG,16, 80 μg/mL) in a 200 μL of immunoprecipitation buffer system (0.5% Triton X-100, 10 mM Tris-HCl, pH 7.5, 150 mM NaCl, 2 mM EDTA-Na_2_). The mixture was incubated with constant rotation for 12 h at 4°C. Protein G-Sepharose beads (P3296; 30 μL/tube; Sigma Aldrich, MO, USA) were added into the system and incubated for 6 h at room temperature. The antigen-antibody complex conjugated with Protein G was separated by centrifugation. The supernatant and the complex were then analyzed by western blotting.

### Transmission Electron Microscopy

Transmission electron microscopy (TEM) was used to observe the morphology of PFF. These samples were diluted to 20 μg/mL with 0.01 M PBS and applied onto 200-mesh copper grids (Agar Scientific, Essex, UK), which were pre-coated with Formvar. About 6 min later, the grids were blotted with a filter paper and negatively stained with 5% uranyl acetate for 3 min. Copper grids were then blotted and dried under infrared radiation for 24 h at 37°C. A JEM-2100 electron microscope (JEOL, Tokyo, Japan) was used to observe the protein structure.

### Thioflavin T Post-Staining

The formation of α-syn fibrils was investigated by detecting the fluorescence intensity of Thioflavin T (ThT) in a diluted mixture of 5 μL × 3 mg/mL protein (monomeric h-α-syn and PFF) and 95 μL × 20 μM of ThT solution. Plates were sealed and incubated at room temperature with fluorescence measurements recorded every 30 min. Excitation and emission wavelengths were fixed at 440 and 485 nm, respectively.

### Mice

Human α-syn (h-α-syn) overexpressing transgenic mice [017682; C57BL/6N-Tg (Thy1-SNCA) 15Mjff/J; hemizygotes] and wild-type (Wt) C57BL mice were purchased from the Jackson Laboratory, Bar Harbor, ME, USA. The α-syn knock-out (KO) mice (C57BL/129X1-Sncatm1Rosl/J) were purchased from the Model Animal Research Center of Nanjing University. The BALB/c, BALB-c/nu mice for antibody productions were bought from Vital River Laboratories (Beijing, China). All animals were housed at room temperature under a 12: 12 h light/dark cycle in the Laboratory Animal Center, Capital Medical University. Animal experiments conformed to the National Institutes of Health (NIH; Bethesda, MD, USA) guidelines for animal care and use. The animal study was reviewed and approved by the Institutional Animal Care and Use Committee. (Capital Medical University Animal Experiments and Experimental Animals Management Committee, AEEI-2014-031).

### Western Blots

Western blotting was performed as previously described (29). Briefly, samples of recombinant protein (m-α-syn/m p-α-syn/h-α-syn/h p-α-syn, 200 ng/lane) and mice brain [midbrain/striatum/cortex homogenates; 30 μg/lane (α-syn detection), 100 μg/lane (p-α-syn detection)] were mixed with a loading buffer (250 mM Tris-HCl, pH 6.8, 30% glycerol, and 0.02% Bromophenol Blue) and then boiled at 95°C for 8 min. Proteins were separated by 12% sodium dodecyl sulfate-polyacrylamide gel electrophoresis. The gels were then transferred to polyvinylidene difluoride membranes (Millipore, MA, USA) at 2 mA/cm^2^ for 90 min. The membranes were incubated with 0.4% paraformaldehyde (PFA) for 30 min and then washed three times with TBST. After being blocked with 5% skimmed milk for 1 h at room temperature, the membranes were incubated with primary antibodies overnight at 4°C. The membranes were then washed three times with TBST and incubated with fluorophore-conjugated secondary antibodies, including mouse 680 (926-68070; LI-COR Biosciences, Lincoln, NE, USA), mouse 800 (926-32210; LI-COR Biosciences, USA), rabbit 680 (926-68071; LI-COR Biosciences, NE, USA) and rabbit 800 (926-32211; LI-COR Biosciences, NE, USA), diluted at 1:10,000. After incubation with secondary antibodies for 1 h, the membranes were washed three times with TBST and visualized in an Odyssey imaging system (LI-COR Biosciences, NE, USA). Primary antibodies used in this study included C140S, p-α-syn#1 (pSyn#64; WAKO, Osaka, Japan), p-α-syn#2 (ab51253; Abcam, Cambridge, USA), α-syn#1 (Sc-69977; Santa Cruz, USA), α-syn#2 (ab138501, Abcam, Cambridge, UK), α-syn#3 (D37A6-4179, Cell Signaling Technology, Danvers, MA, USA), β-actin (66009-1-Ig; Proteintech, Chicago, IL, USA). The details are described in Table 1.

**Table 1:** Antibodies produced and used in this research.

**Name**	**Species**	**Type**	**Immunopeptide sequence**	**Source**
C140S	Mouse	Monoclonal	P1: Ac-CEAYEMP(pS)EGG-NH2, h p-α-syn 123-132	–
C48S	Mouse	Monoclonal	P2: Ac-EMP(pS)EEGYQDC-NH2, h p-α-syn 126-135	–
C54S	Mouse	Monoclonal	P1: Ac-CEAYEMP(pS)EGG-NH2, h p-α-syn 123-132	–
p-α-syn#1	Mouse	Monoclonal	h p-α-syn 124-134	Wako
p-α-syn#2	Rabbit	Monoclonal	h p-α-syn 100-140	Abcam
α-syn#1	Mouse	Monoclonal	-	Santa Cruz Biotechnology
α-syn#2	Rabbit	Monoclonal	h-α-syn 118-123	Abcam
α-syn#3	Rabbit	Monoclonal	surrounding Glu105 of m-α-syn	Cell Signaling technology

*p, phospho-group; h, human; m, mouse*.

### Immunofluorescence

Immunofluorescence assays were conducted as previously described (29). Briefly, the mice were perfused with 0.9% of NaCl in deep anesthesia. After the residual blood was removed, the circulatory system was perfused with 4% of PFA. The brain was removed and then was dehydrated and cryoprotected in 20 and 30% of sucrose solution for 24 h at 4°C, respectively, and then embedded with an optimum cutting temperature (OCT) reagent. The midbrain was cut into 30 μm-thick sections. These sections were immersed in PBST [0.3% Triton X-100 in 0.01 M PBS] for 10 min, followed by incubating in 10% goat serum (5,424; Cell Signaling Technology, MA, USA) for 60 min. C140S, p-α-syn#1 (Wako, Osaka, Japan) and α-syn#2 (Abcam, Cambridge, UK) antibodies were then incubated with sections for 24 h at 4°C. After being washed in PBST three times, the sections were incubated with secondary antibodies [A32744; mouse Alexa Fluor 594 (Invitrogen, Carlsbad, CA, USA), rabbit 488 (A32731; Invitrogen)] for 1 h at room temperature, followed by counterstaining with 4′,6-diamidino-2-phenylindole (D9542; diluted 1:2,000; Sigma-Aldrich, MO, USA) for 15 min. These sections were imaged with a confocal microscope (TCS 473 SP8, Leica, Solms, Germany) for examination.

### PFF and Treatment

A microcentrifuge tube containing 100 μL recombinant h-α-syn (4 mg/mL) was sealed and incubated at 37°C under constant agitation at 1,000 rpm on an Eppendorf Thermomixer Comfort (Eppendorf AG, Hamburg, Germany) for 7 days. The PFFs were examined by TEM and ThT post-staining. The PFF treatment was performed as previously described (30). Primary mouse cortical neurons (wild-type mice) were cultured for 7 days before being treated with h-α-syn monomer and the sonicated-PFF (final concentration: 2 μg/mL). After a single h-α-syn or PFF application, the cells were maintained for 7 days. Neurons were fixed with 4% PFA, and were immersed in PBST [0.3% Triton X-100 in 0.01 M PBS] for 10 min, followed by incubating in 10% goat serum (5,424; Cell Signaling Technology, MA, USA) for 60 min. C140S and p-α-syn#1 (Wako, Osaka, Japan) antibodies were then incubated for immunofluorescence assays.

### Immunohistochemistry of the Amygdala Tissues With p-α-Syn Antibodies

Sections (5 μm thick) of paraffin-embedded amygdala collected by the China National Clinical Research Center for Neurological Diseases (Beijing Tiantan Hospital) were processed for immunohistochemistry. Following dewaxing, hydration, and antigen retrieval, endogenous peroxidase activity was eliminated with 3% of H_2_O_2_ in PBS for 10 min. After washing in PBS, the sections were incubated with 10% goat serum followed by 0.3% TritonX-100 in PBS for 60 min, then incubated with C140S for 24 h at 4°C. For detection, primary antibodies, a biotinylated goat anti-mouse secondary antibody (PV9002; Zhongshan Golden Bridge Biotechnology, Beijing, China), and diaminobenzidine (ZLI-9017; Zhongshan Golden Bridge Biotechnology) were usedto detect the immunoreactivity. The sections were then counterstained with hematoxylin and imaged with a confocal microscope (TCS 473 SP8, Leica) for examination. The studies involving human participants were reviewed and approved by the IRB of Beijing Tiantan Hospital, Capital Medical University (KY2018-031-02).

### Pre-Embedding Immunogold Electron Microscopy With p-α-Syn Antibodies

Mice were perfused with 0.9% of NaCl while in deep anesthesia. After the residual blood was removed, the circulatory system was perfused with 4% of PFA and 0.075% of glutaraldehyde (GA) in 0.1 M PB. The brain was removed and postfixed with 4% of PFA and 0.2% of GA for 8 h. The midbrain was cut into 50 μm-thick sections. These sections were permeabilized with PBST (0.3% Triton X-100 in 0.01 M PBS) for 1 h, followed by incubation with 5% of goat serum (5,425; Cell Signaling Technology) for 1 h at room temperature. The C140S antibody was then incubated with the sections for 24 h at 4°C. After being washed with 1% bovine serum albumin (BSA)-PBST three times, the sections were incubated with secondary antibody conjugated to 1.4 nm gold particles [2002-0.5 mL; Nanogold-Fab goat anti-mouse IgG (H+L); Nanoprobes, Yaphank, NY, USA and 2004-0.5 mL; Nanogold-Fab goat anti-rabbit IgG (H+L); Nanoprobes], diluted 1:50 with 1% BSA-PBST, for 1 h at room temperature. After washing with 1% BSA-PBST three times, the sections were postfixed with 2% GA for 1 h. Silver enhancement (2013; Nanoprobes) was conducted for 5 min, followed by washing with deionized water three times. The immunogold-labeled midbrain sections were then re-embedded for ultrathin sectioning. We used a JEM-2100 electron microscope (JEOL, Tokyo, Japan) to analyze the ultrastructural localization of p-α-syn-specific immunolabeling in the ultrathin sections.

### Statistical Analysis

Prism 8.0.1 software (GraphPad, La Jolla, CA, USA) was used for all statistical analyses. The presented results for each experiment were independently conducted at least three times. The data are expressed as the mean ± standard error of the mean (SEM). Differences of p-α-syn/α-syn levels in mice brain tissues or ThT post-staining between h-α-syn and PFF were evaluated using the unpaired *t*-test. Other data were examined by the One-way ANOVA and Tukey's multiple comparisons tests. In all cases, a value of *P* < 0.05 was considered statistically significant.

## Results

### Generation and Characterization of Recombinant p-α-Syn Protein *in vitro*

**Figure 1:**
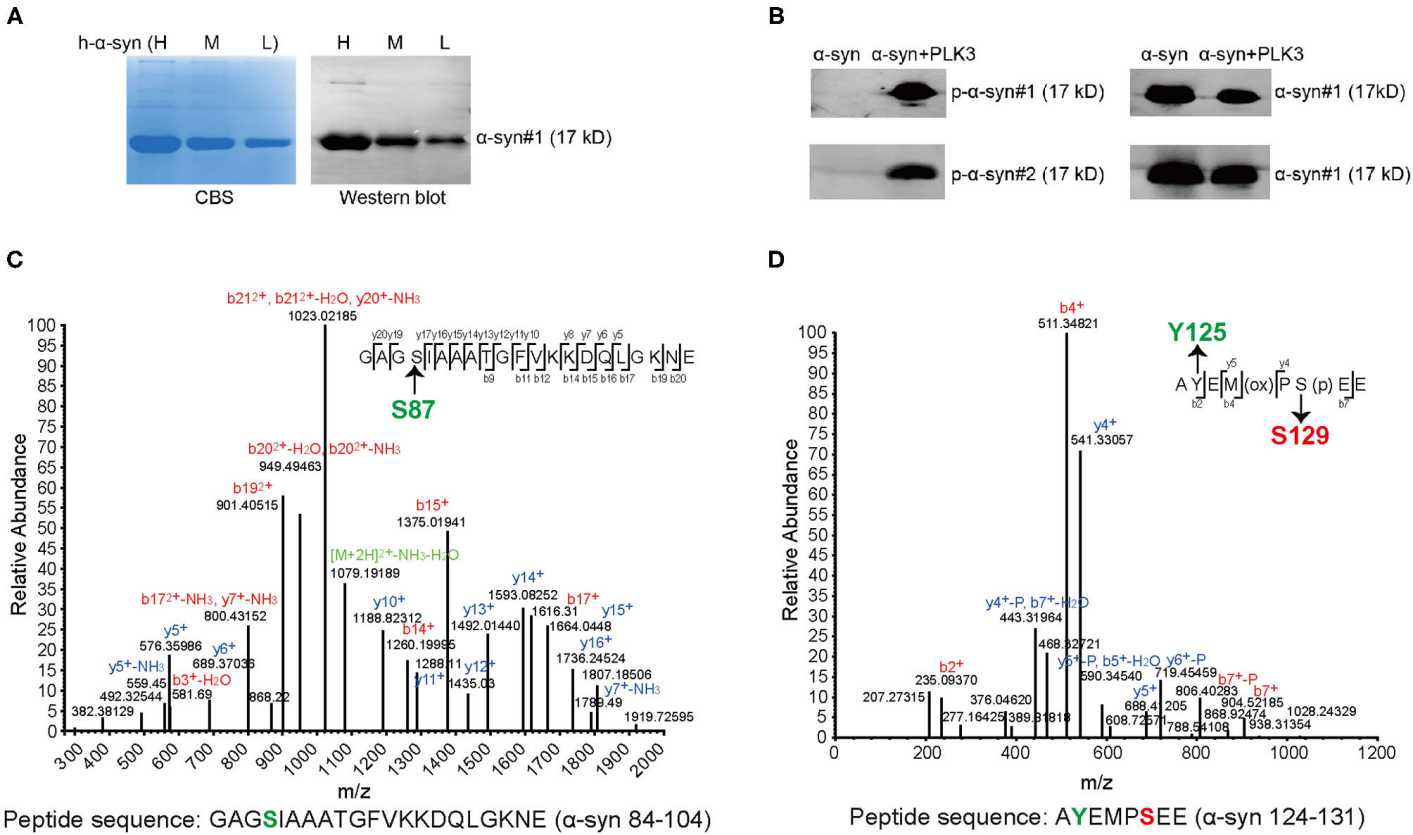
Generation and characterization of recombinant p-α-syn protein *in vitro*. **(A)** Coomassie Brilliant Blue staining (CBS, left) and western blotting (right) of the purified recombinant h-α-syn from *E. Coli*. Three gradient elution with high concentration (H, 3 mg/mL)/moderate concentration (M, 2 mg/mL)/low concentration (L, 1 mg/mL) were analyzed. The h-α-syn was detected with α-syn#1 antibody (Santa Cruz Biotechnology, Santa Cruz, CA, USA). **(B)** Western blotting of 200 ng of recombinant h-α-syn in the absence or presence of Polo-Like-Kinase 3 (PLK3). The p-α-syn was probed with three anti-pSer129 α-syn antibodies (p-α-syn#1 and p-α-syn#2, left blot). The h-α-syn was detected with α-syn#1 antibody (right blot). **(C)** Representative mass spectrometry (MS) identification information of α-syn 84–104. GAGSIAAATGFVKKDQLGKNE (α-syn 84–104) was one of the digested peptides from the resultant p-α-syn (h-α-syn+PLK3). **(D)** Representative MS identification information of α-syn 124–131. AYEMPSEE (α-syn 124–131) was also a digested peptide from the resultant p-α-syn. Detailed MS information is represented in Tables 2, 3. P, phospho-group.

To characterize the specificity of p-α-syn antibodies, we first prepared p-α-syn antigen *in vitro*. We prepared a recombinant p-α-syn protein using the kinase-catalyzed phosphorylation method (28). Recombinant human α-syn (h-α-syn; verified by Coomassie Brilliant Blue staining and western blotting; Figure 1A) was phosphorylated in the presence of Polo-Like-Kinase 3 (PLK3). To further determine whether phosphorylation of α-syn at serine-129 occurred, we used two approaches. First, we used two commercial pSer129 α-syn antibodies to recognize the resulting p-α-syn (p-α-syn#1 and p-α-syn#2). Western blot results (Figure 1B) showed p-α-syn positive bands (17 kD) in the “h-α-syn+PLK3” group, while the band was not detected in the “h-α-syn” group. In parallel, we analyzed the resultant p-α-syn using mass spectrometry (MS) to clearly define the phosphorylation sites of α-syn. The MS results (Figures 1C,D; Tables 2, 3) showed that phosphorylation occurred specifically at amino acid 129 but not at amino acids 87 or 125. These results confirmed that PLK3 specifically phosphorylated α-syn at serine-129, and that we successfully prepared recombinant p-α-syn protein *in vitro*.

**Table 2:** The ion series of peptide AYEMPSEE (α-syn 124–131).

**#1**	**b^**+**^**	**b^**2+**^**	**Sequence**	**y^**+**^**	**y^**2+**^**	**#2**
1	72.04440	36.52584	A	–	–	8
2	235.10772	118.05750	Y	980.29551	490.65139	7
3	364.15032	182.57880	E	817.23219	409.11973	6
4	511.18573	256.09650	M-Oxidation	688.18959	344.59843	5
5	608.23850	304.62289	P	541.15417	271.08072	4
6	775.23686	388.12207	S-Phosphorylation	444.10140	222.55434	3
7	904.27946	452.64337	E	277.10304	139.05516	2
8	–	–	E	148.06044	74.53386	1

**Table 3:** The ion series of peptide GAGSIAAATGFVKKDQLGKNE (α-syn 84–104).

**#1**	**b^**+**^**	**b^**2+**^**	**Sequence**	**y^**+**^**	**y^**2+**^**	**#2**
1	58.02875	29.51801	G	–	–	22
2	129.06587	65.03657	A	2134.11399	1067.56063	21
3	186.08734	93.54731	G	2063.07687	1032.04207	20
4	273.11937	137.06332	S	2006.05540	1003.53134	19
5	386.20344	193.60536	I	1919.02337	960.01532	18
6	457.24056	229.12392	A	1805.93930	903.47329	17
7	528.27768	264.64248	A	1734.90218	867.95473	16
8	599.31480	300.16104	A	1663.86506	832.43617	15
9	700.36248	350.68488	T	1592.82794	796.91761	14
10	757.38395	379.19561	G	1491.78026	746.39377	13
11	904.45237	452.72982	F	1434.75879	717.88303	12
12	1003.52079	502.26403	V	1287.69037	644.34882	11
13	1131.61576	566.31152	K	1188.62195	594.81461	10
14	1259.71073	630.35900	K	1060.52698	530.76713	9
15	1374.73768	687.87248	D	932.43201	466.71964	8
16	1502.79626	751.90177	Q	817.40506	409.20617	7
17	1615.88033	808.44380	L	689.34648	345.17688	6
18	1672.90180	836.95454	G	576.26241	288.63484	5
19	1800.99677	901.00202	K	519.24094	260.12411	4
20	1915.03970	958.02349	N	391.14597	196.07662	3
21	2044.08230	1022.54479	E	277.10304	139.05516	2
22	–	–	E	148.06044	74.53386	1

### Preparation and Selection of Anti-p-α-Syn Mouse Monoclonal Antibodies

**Figure 2:**
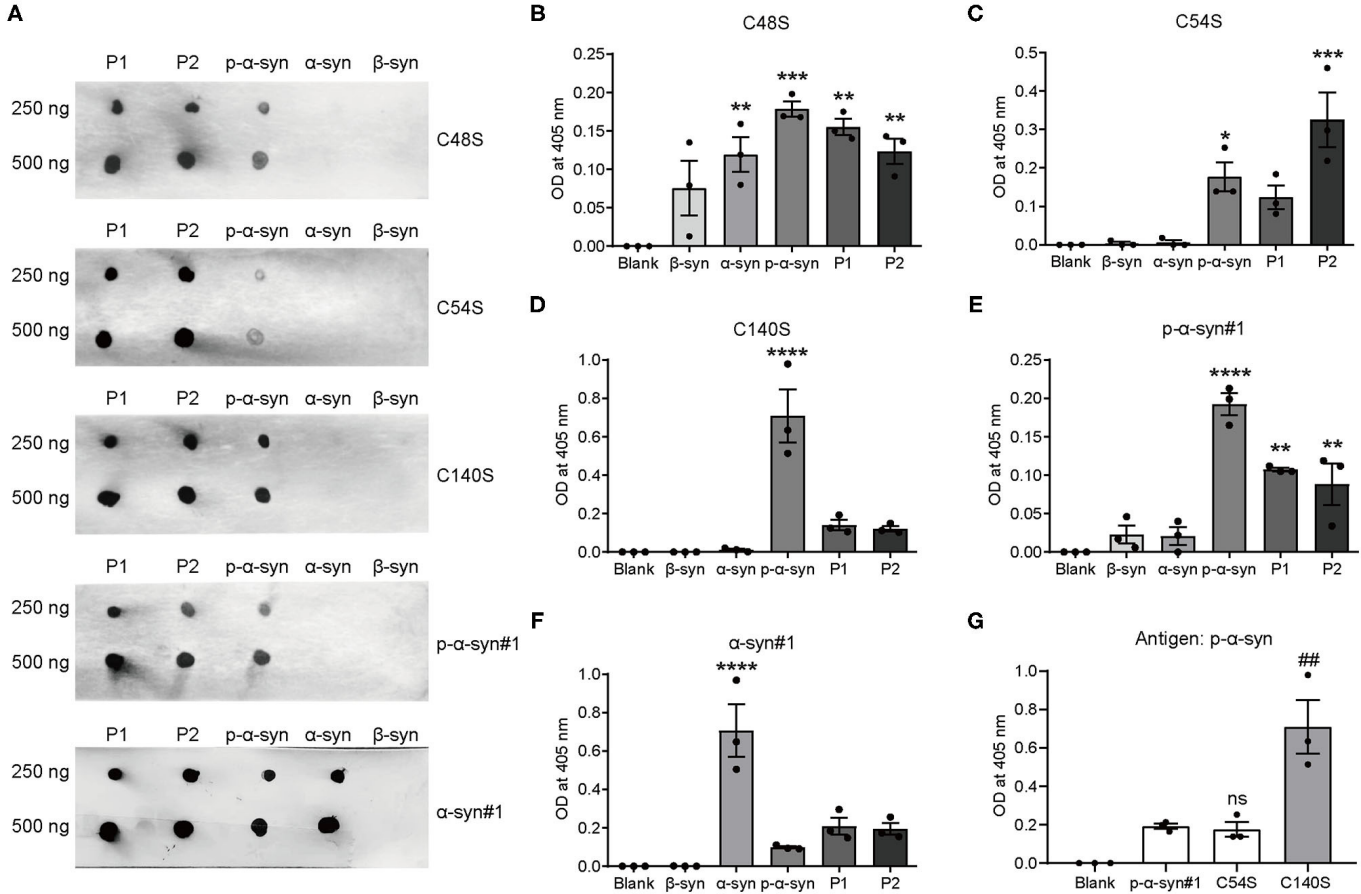
Preparation and selection for anti-p-α-syn mouse monoclonal antibodies. **(A)** In total, 250 and 500 ng of immunopeptides 1 [P1; Ac-CEAYEMP(pS)EGG-NH2; amino acids: 123–132], peptides 2 [P2; Ac-EMP(pS)EEGYQDC-NH2; amino acids: 126–135], p-α-syn, α-syn, and β-syn human proteins were spotted onto nitrocellulose membranes, and then probed with antibodies. The p-α-syn was detected by three screened-out monoclonal antibodies, named as C140S (P1), C48S (P2), and C54S (P1). The p-α-syn#1 (Wako, Osaka, Japan, targeted to amino acids 124–134 of p-α-syn) was used as a positive control. The monoclonal α-syn#1 antibody (Santa Cruz Biotechnology) demonstrated equal loading of proteins. **(B–F)** Quantification of the absorbance differences among C140S, C48S, C54S, p-α-syn#1, and α-syn#1 when recognizing P1, P2, α-syn, p-α-syn, and β-syn (100 μg/mL) by indirect ELISA. **(G)** Quantification of the absorbance difference among C140S, C54S, and p-α-syn#1 when recognizing p-α-syn (100 μg/mL) in indirect ELISAs. The absorbance of plates was measured at 405 nm with a Multiskan MK3 microplate reader. The results are expressed as the mean ± standard error of the mean (SEM) (One-way ANOVA, Tukey's multiple comparisons test, *n* = 3). *P < 0.05, **P < 0.01, ***P < 0.001, ****P < 0.0001 *vs*. Blank; ^##^*P* < 0.01 *vs*. p-α-syn#1. ns, no significance.

Because p-α-syn antibodies with high sensitivity and specificity are crucial for reliable detection, we first prepared and selected anti-p-α-syn mouse monoclonal antibodies peptides (Table 1). We immunized mice with two human p-α-syn peptides, P1 [Ac-CEAYEMP(pS)EGG-NH2; amino acids: 123-132] and P2 [Ac-EMP(pS)EEGYQDC-NH2; amino acids: 126-135]. Among the 17 strains of hybridomas (Table 4), C54S, C48S, and C140S obtained the highest sensitivities. To further test the specificity and affinity of these three p-α-syn antibodies, we conducted dot blot assays (Figure 2A) and indirect ELISAs (Figures 2B–F). The results showed that C54S and C140S exclusively bound to α-syn phosphorylated at the serine-129 residue (P1, P2, and p-α-syn) without cross-reactivity with α-syn and β-synuclein, and the results showed no difference from the commercial p-α-syn#1 antibody (targeted to amino acids 124–134 of p-α-syn). In contrast, C48S cross-reacted with α-syn (Figure 1B). Furthermore, compared to C54S and p-α-syn#1, C140S had much higher specificity toward p-α-syn (Figure 2G). Therefore, we chose C140S (IgG subclasses: IgG2b) for further characterization.

**Table 4:** Sensitivity of p-α-syn mouse monoclonal antibodies secreted from hybridomas.

**Name**	**Volume (mL)**	**Theoretical sensitivity (ng)**	**Dilution ratio**	**Volume in use (μL)**
C16S	0.1	0.05	1:500	10
C144S	0.1	0.05	1:500	10
C55D	0.15	5	1:100	50
**C48S**	**0.1**	**0.01**	**1:500**	**10**
C104S	0.1	0.25	1:250	20
C113D	0.15	0.01	1:500	10
C161S	0.1	0.05	1:500	10
C141D	0.15	5	1:100	50
C23S	0.15	0.05	1:500	10
C142S	0.1	0.01	1:500	10
**C140S**	**0.1**	**0.01**	**1:500**	**10**
C37S	0.1	0.05	1:500	10
C78S	0.1	0.01	1:500	10
**C54S**	**0.1**	**0.01**	**1:500**	**10**
C57D	0.1	5	1:100	50
C95D	0.1	5	1:100	50
C86S	0.1	1	1:200	25

*Bold fonts indicate our selected monoclonal antibodies*.

### Specificity and Affinity of the C140S Antibody

**Figure 3:**
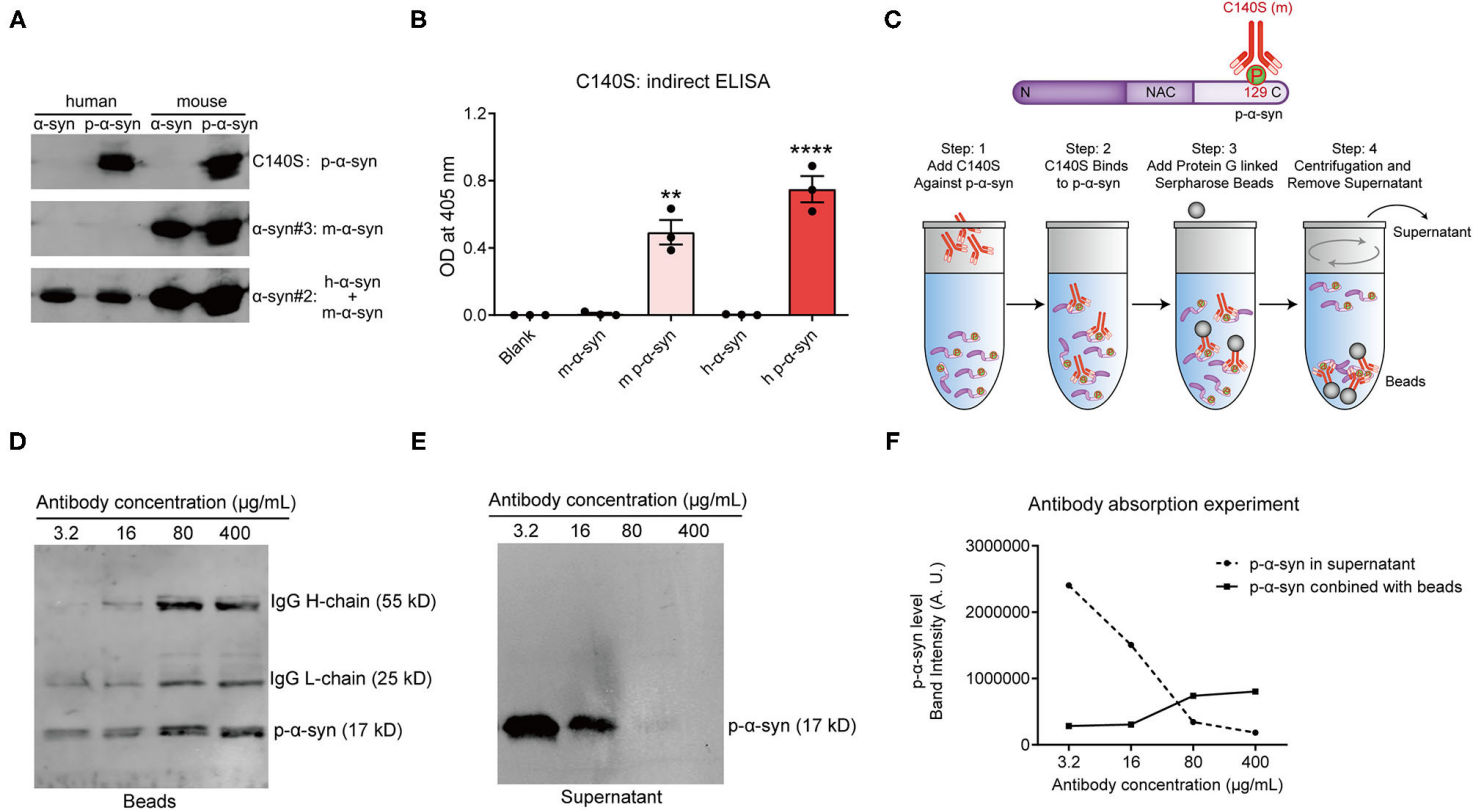
Determining the specificity and affinity of C140S. **(A)** A total of 200 ng of human p-α-syn (h p-α-syn) and mouse p-α-syn (m p-α-syn) were prepared and probed by western blots with C140S. The α-syn#2 antibody (Abcam, Cambridge, UK) was used to detect human α-syn. The α-syn#3 antibody (CST) demonstrated an equal loading of mouse α-syn. **(B)** The affinity of C140S was assessed using indirect ELISAs on m-α-syn/m p-α-syn/h-α-syn/h p-α-syn protein (100 μg/mL) coated wells. The absorbance of plates was measured at 405 nm with a Multiskan MK3 microplate reader. The results are expressed as the mean ± standard error of the mean (SEM) (One-way ANOVA, Tukey's multiple comparisons test, *n* = 3). **P < 0.01, ****P < 0.0001 *vs*. Blank. **(C)** The schematics of the antibody absorption experiment. N, N-terminal domain of α-syn (residues 1–60). NAC, non-amyloid component, is the central domain of α-syn (residues 61–95). C, C-terminal domain of α-syn (residues 96–140). P, phospho-group. m, mouse. According to the experimental schematics, 5 μg h of p-α-syn was incubated with different concentrations of C140S antibody (3.2, 16, 80, and 400 μg/mL). The protein G beads were added to conjugate with the antigen-antibody complex. The beads-antigen-antibody complex and supernatant were separated by centrifugation and examined using western bolts, shown in **(D,E)**. The p-α-syn was detected with p-α-syn#1 antibody (Wako, Osaka, Japan). **(F)** Quantification of the band intensities of p-α-syn in the beads-antigen-antibody complex **(D)** and supernatant **(E)**.

It was verified that C140S had high specificity toward human p-α-syn (h p-α-syn), when using an indirect ELISA (Figure 2D). To further verify whether C140S recognized mouse p-α-syn (m p-α-syn), we prepared m p-α-syn and analyzed it with C140S. Western blotting (Figure 3A) and an indirect ELISA (Figure 3B) showed that C140S reacted with both h p-α-syn and m p-α-syn proteins. To further test the affinity of C140S toward p-α-syn, we conducted the following antibody absorption experiment, a brief summary of which is shown in the schematic diagram (Figure 3C). The results (Supplementary Figures S2A,B) showed that C140S, but not the control mouse IgG, specifically bound to p-α-syn, and as the additional amount of C140S increased, the enriched p-α-syn increased (Supplementary Figures S2B,C). Further experiments (Figures 3D–F) showed that the amount of p-α-syn (17 kD) in the supernatant decreased gradually with increasing concentrations of C140S, whereas p-α-syn in the complex increased gradually with the increasing concentrations of C140S. Complete depletion of p-α-syn in the supernatant was achieved when C140S concentration reached 400 μg/mL. Together, these results showed that C140S had high specificity and affinity for p-α-syn protein.

### Comparative Immunoblotting of p-α-Syn Using C140S and p-α-Syn Commercial Antibodies

**Figure 4:**
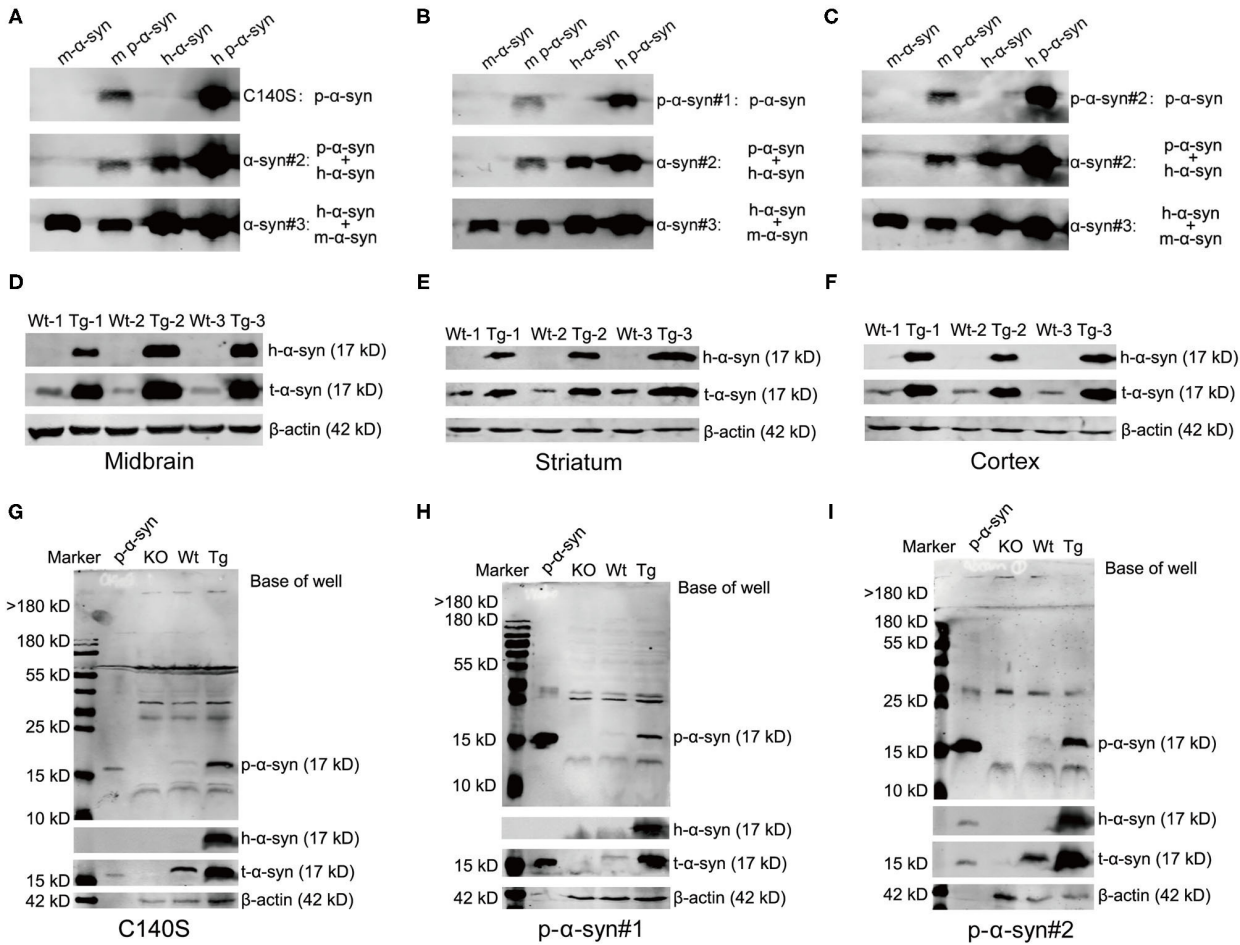
Western blot results showed that C140S had similar recognition patterns as commercial p-α-syn antibodies. **(A–C)** Comparative immunoblotting of p-α-syn protein (prepared *in vitro*) between C140S, p-α-syn#1, and p-α-syn#1. A total of 200 ng m-α-syn/m p-α-syn/h-α-syn/h p-α-syn proteins were denatured and analyzed with C140S **(A)**, p-α-syn#1 [Wako, Osaka, Japan, **(B)**], and p-α-syn#2 [Abcam, Cambridge, UK, **(C)**]. The h-α-syn was detected with α-syn#2 antibody (Abcam, Cambridge, UK). The m-α-syn was detected with α-syn#3 antibody (CST). D-F. A total of 30 μg of brain homogenates of the midbrain **(D)**/the striatum **(E)**/the cortex **(F)** from wild-type brood (Wt)/Thy1-SNCA transgenic (Tg) mice were denatured and analyzed with h-α-syn and α-syn antibodies. **(G–I)** Comparative immunoblotting of mice midbrain between C140S, p-α-syn#1, and p-α-syn#2. 100 μg of midbrain homogenates from α-syn knock-out (KO)/Wt/Tg mice were denatured and analyzed with C140S **(G)**, p-α-syn#1 **(H)**, and p-α-syn#2 **(I)**. The h-α-syn was recognized by α-syn#2 antibody (Abcam, Cambridge, UK), and was used to show the successful overexpression of h-α-syn in the brain of Tg mice. The total α-syn level was detected with α-syn#1 antibody (Santa Cruz, CA, USA). The p-α-syn protein was added as a positive control. The KO mice were used as a negative control. β-actin (42 kD) was used as a loading and internal control to enable sample normalization. Mouse age: 13-months-old.

To better characterize the specificity of C140S, we compared C140S with commercially available p-α-syn antibodies, and analyzed their recognition patterns toward p-α-syn, both *in vitro* and *ex vivo*. First, we denatured and analyzed 200 ng of the m-α-syn/m p-α-syn/h-α-syn/h p-α-syn proteins with C140S (Figure 4A), p-α-syn#1 (Wako, Osaka, Japan; Figure 4B), and p-α-syn#2 (Abcam, Cambridge, UK; Figure 4C). The western blot results showed that C140S specifically recognized m-p-α-syn/h p-α-syn rather than m-α-syn/h-α-syn, in a similar manner to p-α-syn#1 and p-α-syn#2 antibodies. We further demonstrated the specificity of C140S utilizing the brains of Thy1-SNCA transgenic (Tg) mice, which overexpressed h-α-syn driven by a broad neuronal promoter. We measured the expression level of α-syn using midbrain/striatum/cortex homogenates from 13-month-old Tg mice. Western blot results (Figures 4D–F; Supplementary Figures S1A–C) showed that α-syn was overexpressed to a moderate degree (two- to five-fold) in the brain regions examined in Tg mice when compared with their wild-type (Wt) littermates, indicating that this Tg mouse model could mimic the familial forms of PD. On this basis, we detected p-α-syn levels in the midbrain of Tg mice, using C140S and two commercially available p-α-syn antibodies (p-α-syn#1 and p-α-syn#2). Immunoblotting results of α-syn KO mice/Wt/Tg midbrains showed that C140S (Figure 4G) detected a band near the expected size of p-α-syn protein (17 kD), which was absent in the midbrain samples of KO mice, showing no differences with p-α-syn#1 (Figure 4H) and p-α-syn#2 (Figure 4I). It should be noted that these p-α-syn antibodies partially cross-reacted with other protein bands near 30–60 kD, which also existed in the KO mice. Moreover, we also found that the levels of p-α-syn (17 kD) were significantly increased in the midbrain homogenates of Tg mice (13-month-old), when compared to the controls (Supplementary Figure S1D). Based on these observations, we next used C140S to detect α-syn pathologies in the brains of Tg mice.

### Immunofluorescence Staining of p-α-Syn in the Midbrain of Tg Mice

**Figure 5:**
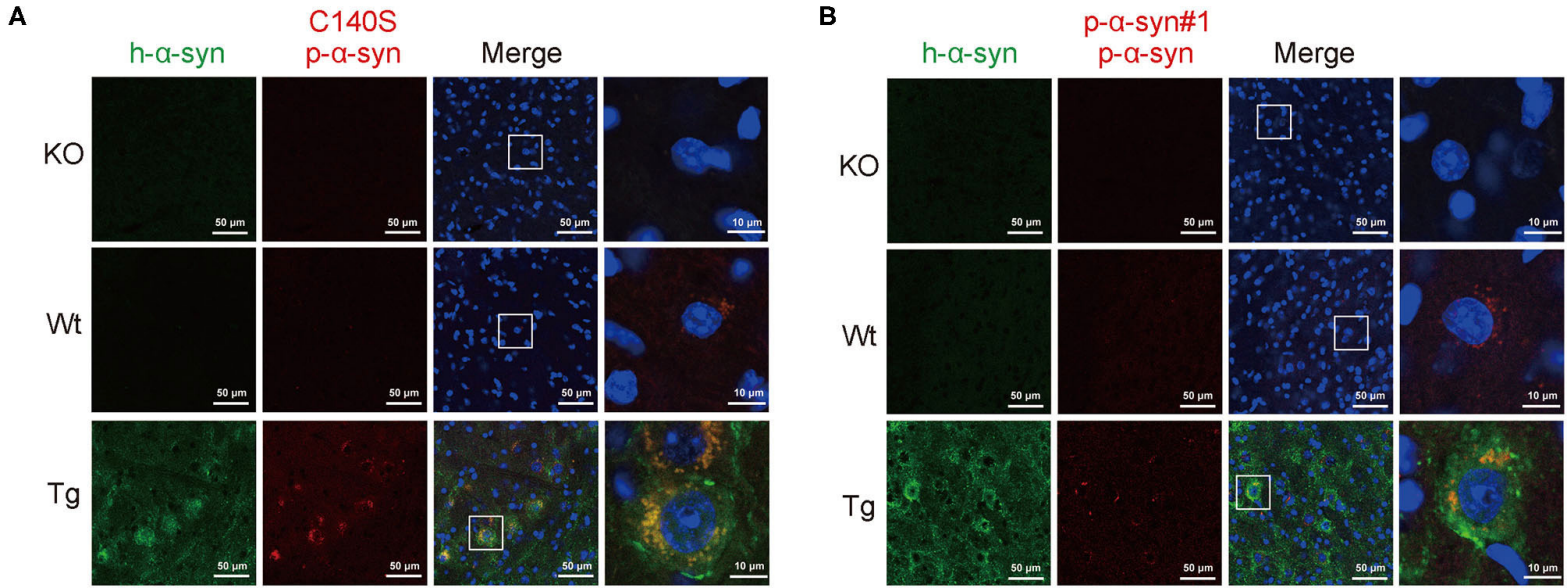
Immunofluorescence staining of p-α-syn in the midbrain of Tg mice using C140S and p-α-syn#1. **(A,B)** The midbrain sections from α-syn knock-out (KO)/Wild-type brood (Wt)/Thy1-SNCA transgenic (Tg) mice were prepared and analyzed with C140S **(A)** and p-α-syn#1 **(B)**. The h-α-syn (green) recognized by α-syn#2 antibody (Abcam, Cambridge, UK) was used to show the successful overexpression of h-α-syn in the midbrain of Tg mice. Red color indicates p-α-syn staining (C140S) and blue color indicates nucleus. White squares, amplified area of midbrain section under low magnification. Scale bar, 50 μm (low magnification) and 10 μm (high magnification). Mouse age: 13-months-old.

To test the synuclein pathological features in the midbrain of Tg mice, we prepared the midbrain sections from 13-month-old KO/Wt/Tg mice, and analyzed p-α-syn with C140S and p-α-syn#1 using immunofluorescence staining. Figure 5A shows that in the midbrain sections from α-syn KO mice, C140S did not produce a detectable signal. We also found that C140S produced a similar staining pattern to the commercial p-α-syn#1 antibody (Figure 5B). In addition, the level of p-α-syn was higher in Tg than in the Wt mice. Moreover, immunostaining results using high magnification showed that p-α-syn was mainly distributed in the cytoplasm, and that most of the p-α-syn was aggregated.

### Immunogold Ultrastructural Observations of p-α-Syn in the Midbrain of Tg Mice

**Figure 6:**
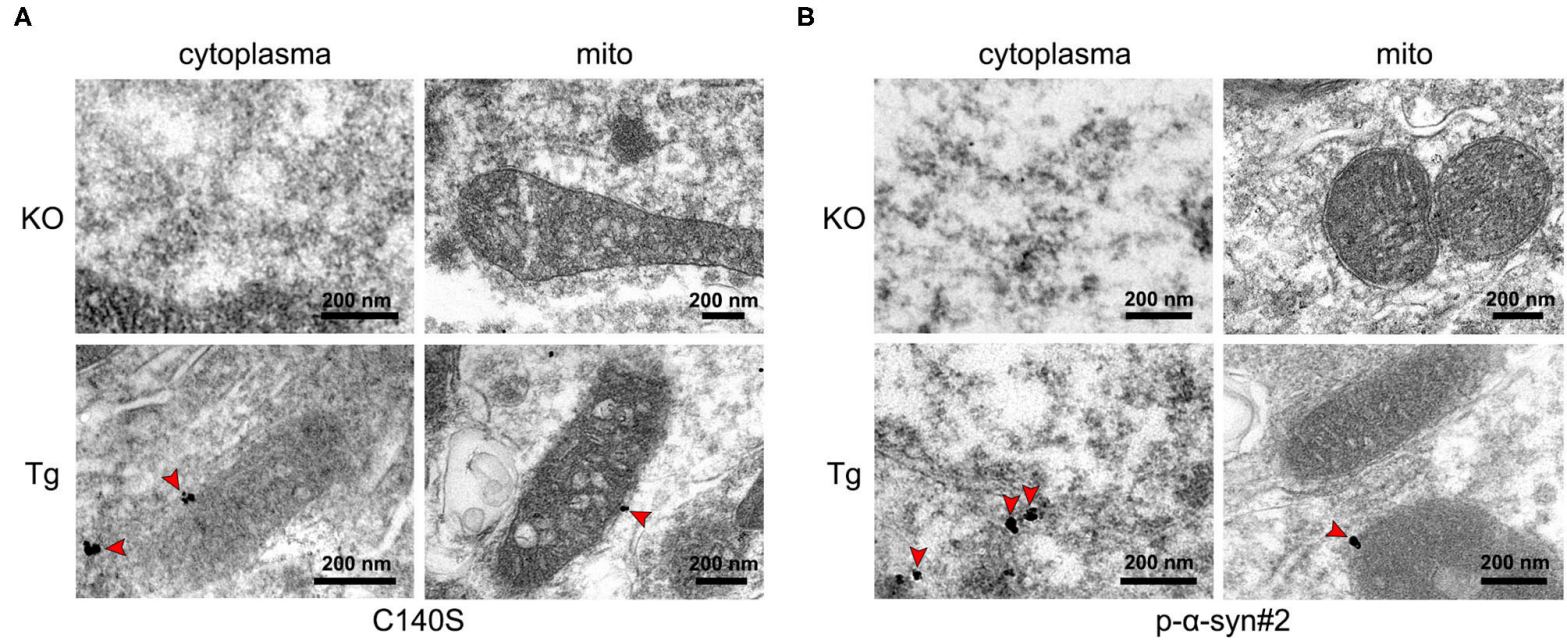
Electron microscopy with immunogold labeling of p-α-syn in the midbrains of Tg mice using C140S and p-α-syn#2. **(A,B)** Immunogold labeling of C140S **(A)** and p-α-syn#2 **(B)** in the midbrain of α-syn knock-out (KO) mice and Thy1-SNCA transgenic (Tg) mice. The p-α-syn was specifically deposited in the cytoplasm, and colocalized with the outer mitochondrial membrane of Tg mice. Red arrow, the concentrated region of p-α-syn. Scale bar, 200 nm. Mice age: 13-months-old.

To determine the subcellular localization of p-α-syn aggregates, the Tg midbrain (13-months-old) was fixed and labeled with C140S and p-α-syn#2 for pre-embedding in the immunogold electron microscopy. The KO mice were also included as negative controls in the following tests. The results (Figure 6A) showed that the labels for C140S were specifically localized in the cytoplasm, and colocalized with the outer mitochondrial membrane, which was consistent with the results of p-α-syn#2 (Figure 6B). These results suggested that p-α-syn might play a role in the mitochondrial dysfunction.

### Immunofluorescence Staining of p-α-Syn Pathological Inclusions in the PFF-Treated Neurons

**Figure 7:**
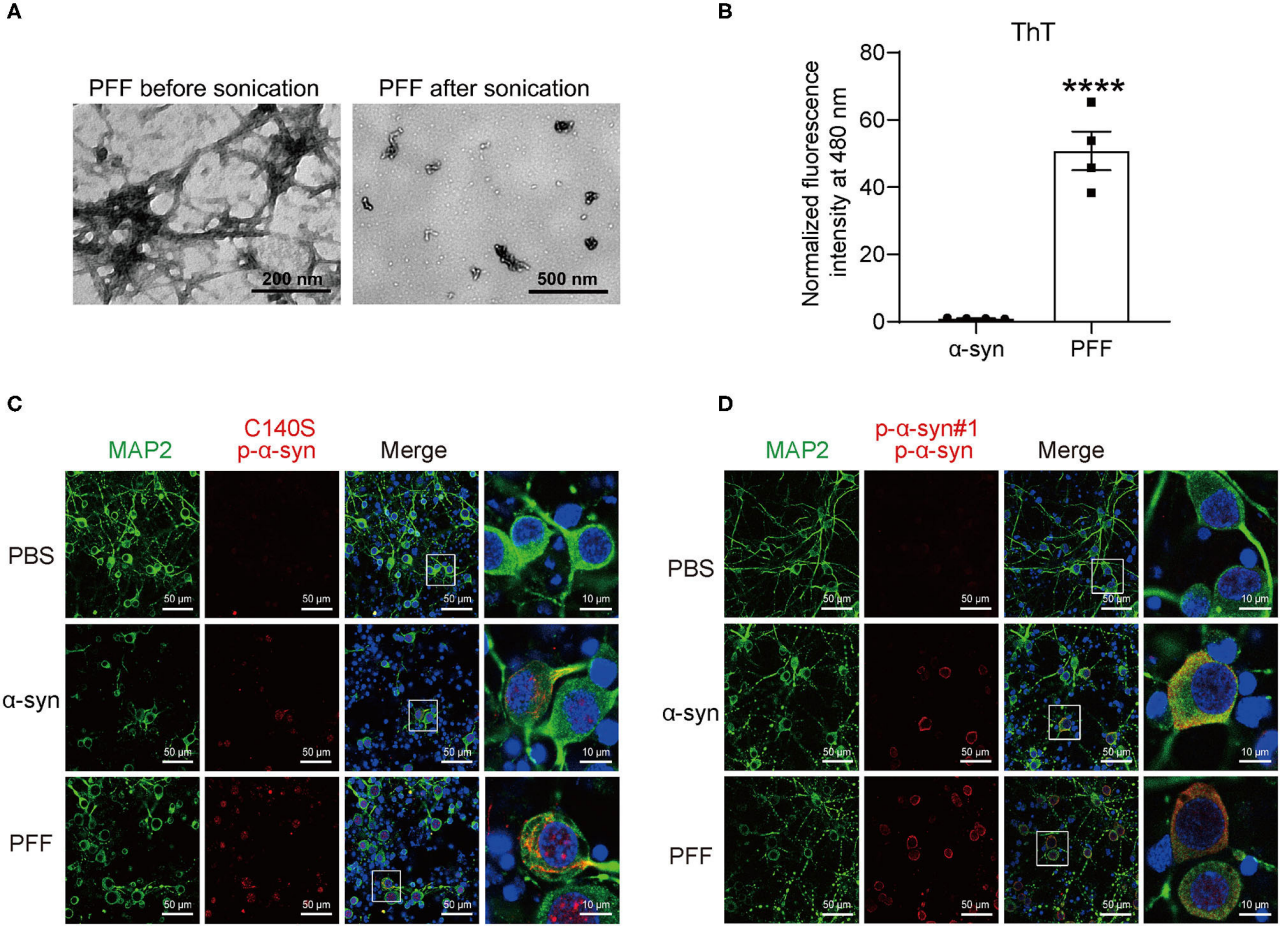
Immunofluorescence staining of p-α-syn in primary neurons using C140S and p-α-syn#1. **(A)** Representative electron microscopy images of preformed fibrils (PFF, left) and the sonicated PFF (right). About 200 ng of PFF (before or after sonication) were applied onto 200-mesh copper grids. Scale bar, 200/500 nm. **(B)** Thioflavin T (ThT) post-staining of monomeric h-α-syn and PFF. **(C,D)** Immunofluorescence staining of p-α-syn in the cortical neurons of the primary mouse showed pathological inclusions. Primary neurons were treated with PBS, monomeric h-α-syn, and sonicated PFF, using C140S **(C)** and p-α-syn#1 **(D)**. Green color indicates microtubule-associated protein 2 (MAP2, green), red color indicates p-α-syn staining (C140S and p-α-syn#1), and blue color indicates nucleus. White squares, amplified area of neurons under low magnification. Scale bar, 50 μm (low magnification) and 10 μm (high magnification). The results are expressed as the mean ± standard error of mean (SEM) (unpaired *t-*test, *n* = 4). ****P < 0.0001 *vs*. α-syn.

It was reported that the addition of exogenous α-synuclein PFFs to primary neuronal cultures could induce the recruitment of endogenous α-synuclein to LB and LN-like aggregates (30–32). To further examine the pathological inclusions in the neurons induced by PFFs, we first generated PFFs by physically shaking monomeric h-α-syn. To prove that aggregation of α-syn occurred, PFFs were determined by negative-staining transmission electron microscopy (TEM). The results (Figure 7A) showed that α-syn aggregates were successfully formed in the PFF group. We also analyzed the fibrillar structure of PFFs using ThT post-staining. The results showed that the fluorescence of PFF aggregates was higher than α-syn monomers (Figure 7B). Together, the results showed that we successfully prepared PFF.

Primary mouse cortical neurons were prepared from E16-18 embryonic brains, and maintained for 7 days *in vitro*, following treatment with either phosphate-buffered saline (PBS) vehicle control, monomeric h-α-syn, or PFF, for 7 days. PFFs were sonicated to lengths of 40–120 nm (examined by TEM) before adding them into the medium. The results of p-α-syn immunofluorescence staining with C140S showed (Figure 7C) that when compared with monomeric h-α-syn treated cultures, the PFF-treated neurons showed higher levels of p-α-syn, with similar results also observed using p-α-syn#1 (Figure 7D). Notably, p-α-syn aggregates showed more punctate and serpentine structures within cell bodies stained with C140S (Figure 6C) while the p-α-syn#1-stained cells showed diffusely distributed p-α-syn (Figure 7D). Moreover, we also found the presence of p-α-syn aggregates in the nucleus of α-syn/PFF treated neurons, while nothing was detected in neurons treated with PBS.

### C140S Detected p-α-Syn Histopathological Features in the Amygdala of Patients With PD

**Figure 8:**
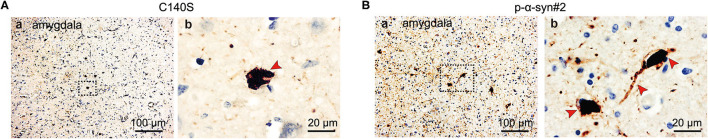
Immunohistochemical staining of p-α-syn in the brain tissue of patients with PD using C140S and p-α-syn#2. Representative images of the immunoreactive structures detected with C140S **(A)** and p-α-syn#2 **(B)** in the amygdala of one patient with PD (male; 69 years old; Braak PD stage V). The brown color indicates p-α-syn staining (C140S and p-α-syn#2) and the blue color indicates hematoxylin (nucleus). Blank square (b), the amplified area of section in low magnification (a). Red arrow, the concentrated region of aggregated p-α-syn. Scale bar, 100 and 20 μm.

To further determine the ability of C140S to recognize disease-relevant pathological lesions in the post-mortem human brains, we obtained 5 μm thick amygdala sections from one patient with PD (male; 69-years-old; Braak PD stage V, collected by Beijing Tiantan Hospital), who was clinically diagnosed and pathologically confirmed with PD. We conducted immunohistochemistry using C140S, which showed that C140S recognized intracytoplasmic LBs and long thin LNs in these sections (Figure 8A), which was consistent with the results of p-α-syn#2 (Figure 8B).

## Discussion

The detection of p-α-syn heavily relies on the specificity of antibody. In the current study, we generated a series of mouse monoclonal p-α-syn antibodies, and selected the C140S antibody. The C140S reacted with the original immunopeptide P1 [Ac-CEAYEMP(pS)EGG-NH2; amino acids: 123–132], and also specifically recognized p-α-syn prepared *in vitro* and *ex vivo*, without cross-reacting with unphosphorylated α-syn and β-syn. Compared with commercial p-α-syn antibodies, in multiple experimental assays, C140S showed similar specificities toward p-α-syn. Therefore, we used C140S to examine α-syn pathologies in PFF-treated neurons, the midbrain of Thy1-SNCA transgenic mice, and in the amygdala of patients with PD. We also observed the ultrastructural localization of p-α-syn in mitochondria with C140S by immunogold electron-microscopy.

A widely used approach for the production of peptide antibodies is to immunize animals with a synthetic peptide, especially for raising antibodies against posttranslational modifications (33). In the current study, we used this experimental biology to generate a series of mouse monoclonal p-α-syn antibodies, and selected C48S, C54S, and C140S for further validation. Although the peptides we used for immunization were 11 amino acids (8–20 amino acids are recommended), we were still not sure whether C48S, C54S, and C140S antibodies could recognize the full-length p-α-syn antigens in denatured or native forms. Dot blot results (Figure 2A) showed that C48S, C54S, and C140S all recognized P1/P2 peptides, while compared to C48S and C54S, C140S showed better substrate recognition toward native full-length p-α-syn antigens. It was reported that the addition of a phosphate group to serine-129 altered intramolecular interactions with the C-terminus of α-syn (34). The environment, such as pH, calcium, and salt also affected the physiological structure of α-syn (3). In the present study, we prepared recombinant p-α-syn protein using the kinase-catalyzed phosphorylation method, and the reaction was performed in a working buffer. In contrast, P1/P2 peptides were dissolved in PBS. We proposed that phosphorylation reaction and the difference of solution might influence the secondary structure of p-α-syn. That might be the reason for the differential recognition of C48S and C54S toward P1/P2 peptides and p-α-syn.

Both western blotting and ELISA results showed that C140S specifically recognized m-p-α-syn/h p-α-syn rather than m-α-syn/h-α-syn, which showed no difference from the commercial p-α-syn#1 and p-α-syn#2 antibodies. We further tested the specificity of C140S in the midbrain of Tg mice by western blotting. Our prior results showed that aged Tg mice had higher levels of aggregated α-syn and p-α-syn in the soluble and insoluble components of the striatum (35). Consistent with these results, using C140S, we detected increased levels of p-α-syn (17 kD) in the midbrain of Tg mice (13-months-old). We also examined the histopathological features in the amygdala of patients with PD. Filamentous aggregates of p-α-syn were found in the axons and soma of neurons, which meant that C140S recognized intracytoplasmic LBs and long thin LNs. Taken together, the C140S showed relatively high specificity toward p-α-syn prepared both *in vitro* and *ex vivo*. Our present results showed that C140S specifically recognized full-length p-α-syn antigens (prepared *in vitro*) in native forms [Dot blot results (Figure 2A) and indirect ELISA results (Figures 2D, 3B)] and denatured forms [Western blot results (Figures 3A, 4A)]. Warranting caution, it was reported that different methods of preparing the tissues, such as different antigen retrieval methods and blocking agents might affect p-α-syn antibody performance. The p-α-syn#2 was found to detect an off-target higher-molecular weight protein unrelated to α-syn, but this protein was apparently not detected *via* immunohistochemistry or confocal approaches (27). Consistent with this, our immunofluorescence staining results (Figure 5A) showed that C140S and p-α-syn#1 specifically recognized p-α-syn antigens (mouse brain) in native forms, without cross-reaction with other brain proteins in α-syn KO mice. In contrast, western blot results showed that C140S and the other two commercial and p-α-syn#2 antibodies partially cross-reacted with denatured protein bands near 30–60 kD, which also existed in KO mice (Figures 4G–I). We considered the reason for this difference in the recognition of mouse brain in native forms and denatured forms might be due to the states or structural changes of brain proteins after heat-denatured in the presence of SDS and DTT. The denaturation of some proteins in the mouse brain results in the exposure of some similar epitopes, which might cause the cross-reaction of C140S, p-α-syn#1, and p-α-syn#2 antibodies. Previous studies had reported that the pSer473 neurofilament light epitope contained some sequence identity to pSer129 α-syn (26), we will further test whether C140S cross-react with pSer473 neurofilament light antigens in native forms and denatured forms. We also plan to excise, extract, and analyze these nonspecific bands in midbrain of α-syn KO mice by LC-MS/MS.

Even though a large number of studies have shown that aggregated α-syn partly localized to the mitochondria under pathological states, and α-syn accumulation and mitochondrial dysfunction have been implicated in the pathology of PD (36–41, 48), the mechanisms by which p-α-syn and mitochondrial proteins regulate each other to trigger mitochondrial dysfunction are poorly understood. Our p-α-syn electron microscopy immunogold results showed that p-α-syn partly localized to the mitochondria in the midbrain of 13-months-old Thy1-SNCA transgenic mice (Figures 6A,B), suggesting the possibility of direct interaction between p-α-syn and mitochondrial proteins. One previous study supports this possibility. They used the combination of peptide pulldown assays and mass spectrometry to identify the interaction proteins of p-α-syn in the mouse brain synaptosomes, and found that the biotinylated pS129 peptides interacted with some mitochondrial proteins, such as solute carrier family 25 and serine-lactamase-like protein, LACTB (42). Another relevant study by Diego Grassi et al. showed that they discovered the existence of a phosphorylated α-syn species, and this type of p-α-syn aggregates was associated with mitochondria, and induced mitochondrial toxicity and fission, energetic stress, and mitophagy (43). Overall, the exact mechanism for these interactions between p-α-syn and mitochondrial proteins still needs more in-depth research. Additional studies will be necessary to identify the mitochondrial proteins that interacted with p-α-syn in the midbrain of Tg mice by Co-IP/MS, and to clarify the modulatory roles of p-α-syn in mitochondria.

Following the immunofluorescence staining experiments, we found an interesting phenomenon, monomeric h-α-syn, and PFF treatment caused α-syn pathology not only in the cytosol but also in the nucleus (Figures 7C,D). Despite being α-syn partially localized in the nucleus, the reason for its phosphorylation and abnormal aggregation in the nucleus under pathological situations remains unclear (44, 45). Pinho et al. reported that the accumulation of α-syn species in the nucleus altered gene expression and reduced the toxicity of H4 cells, and the effect on gene expression and cytotoxicity was also modulated by phosphorylation on serine 129 site (46). A recent study also found an association between α-syn and DNA. Sydney E.Dent et al. used a combination of electrophoretic mobility shift assay and atomic force microscopy approaches, and found both α-syn and p-α-syn directly bind DNA within the major groove, and p-α-syn could induce the reductions in the ability of α-syn to bind and bend DNA, which meant that abnormally elevated p-α-syn might modulate DNA metabolism (47). Collectively, all these findings above support the potential modulatory roles of p-α-syn in the nucleus.

In summary, the best way to determine the role of p-α-syn is by using a highly specific antibody. We therefore generated and characterized a novel mouse monoclonal p-α-syn antibody named, C140S, in the present study, and a variety of validation assays were conducted to demonstrate the specificity of this antibody. The C140S could reliably detect α-syn pathologies in the cells and mice models of PD, and the brains from patients with PD. Overall, the antibody described herein should serve as a useful tool in the detection and mechanistic studies of pathogenic p-α-syn in PD and related pathologies.

## Data Availability Statement

The raw data supporting the conclusions of this article will be made available by the authors, without undue reservation.

## Ethics Statement

The studies involving human participants were reviewed and approved by IRB of Beijing Tiantan Hospital, Capital Medical University (KY2018-031-02). The patients/participants provided their written informed consent to participate in this study. The animal study was reviewed and approved by Capital Medical University Animal Experiments and Experimental Animals Management Committee, AEEI-2014-031. Written informed consent was obtained from the individual(s) for the publication of any potentially identifiable images or data included in this article.

## Author Contributions

WL and HY conceived the manuscript. WL, QZ, and HX conducted experiments. WL wrote the manuscript. JL, GG, and HY reviewed the manuscript. YH kindly provided the brain sections of PD patients. HY was responsible for ensuring that the descriptions were accurate and agreed by authors.

## Funding

This work was supported by the National Natural Science Foundation of China (No. 81870994), the National Natural Science Foundation of China (No. 82071417), and the National Key Plan for Scientific Research and Development of China (No. 2016YFC1306000).

## Conflict of Interest

The authors declare that the research was conducted in the absence of any commercial or financial relationships that could be construed as a potential conflict of interest.

## Publisher's Note

All claims expressed in this article are solely those of the authors and do not necessarily represent those of their affiliated organizations, or those of the publisher, the editors and the reviewers. Any product that may be evaluated in this article, or claim that may be made by its manufacturer, is not guaranteed or endorsed by the publisher.

## Supplementary Material

Supplementary Figure S1 Quantification of band intensities of α-syn and p-α-syn in the brains of Wt/Tg mice. **(A–C)** Quantification of the band intensities of α-syn levels in the midbrain **(A)**/striatum **(B)**/cortex **(C)** from wild-type brood (Wt) and Thy1-SNCA transgenic (Tg) mice (Figures 4D–F). **(D)** Quantification of the band intensities (17 kD) of p-α-syn levels in the midbrains of Wt and Tg mice (Figures 4G–I). The results are expressed as the mean ± standard error of mean (SEM) (unpaired *t-*test, *n* = 3–10). ^*^*P* < 0.05, ^***^*P* < 0.001 *vs*. Wt. Mice age: 13-months-old.

Supplementary Figure S2 The specificity and affinity of C140S were confirmed by antibody absorption experiment. **(A,B)** A total of 5 μg h of p-α-syn was incubated with control mouse IgG (m-IgG) and C140S antibody (16 μg/mL). The protein G beads were added to conjugate with the antigen-antibody complex. The beads-antigen-antibody complex and supernatant were separated and examined using western bolts, shown in **(A,B)**. The p-α-syn was detected with p-α-syn#2 antibody (Abcam, Cambridge, UK), m-IgG was detected with fluorophore-conjugated secondary antibody mouse 680 (m680). **(C,D)** A total of 5 μg h of p-α-syn was incubated with m-IgG and C140S antibody (16, 80 μg/mL). The protein G beads were added to conjugate with the antigen-antibody complex. The beads-antigen-antibody complex was separated and examined using western bolts, shown in **(C,D)**. The p-α-syn was detected with p-α-syn#2 antibody (Abcam, Cambridge, UK), m-IgG was detected with m680. The p-α-syn protein was added as a positive control. Ab, Antibody.

## Abbreviations

α-syn: α-synuclein
AP: alkaline phosphatase
β-syn: β-synuclein
BCA: bicinchoninic acid
C: C-terminal domain of α-synuclein (residues 96–140)
CBS: Coomassie Brilliant Blue staining
ELISA: enzyme-linked immunosorbent assay
h-α-syn: human α-synuclein
m-α-syn: mouse α-synuclein
MS: mass spectrometry
N, N-terminal domain of α-synuclein (N-α-syn: residues 1-60)
ΔN: ΔN-terminal domain of α-synuclein
NAC, non-amyloid component: the central domain of α-synuclein (residues 61-95)
p: phospho-group
p-α-syn: serine-129-site phosphorylated α-synuclein
PD: Parkinson's disease
PFF: preformed fibrils
PLK3: Polo-Like-Kinase 3
pNPP: p-nitrophenyl phosphate
KO: α-syn knock-out mice
R: rabbit
SD: standard deviation
SEM: standard error of the mean
TEM: transmission electron microscopy
Tg: Thy1-SNCA transgenic mice
ThT: thioflavin T
Wt: wild-type brood mice.

## References

[1] Goedert M Spillantini MG Del Tredici K Braak H. 100 years of Lewy pathology. Nat Rev Neurol. (2013) 9:13–24. 10.1038/nrneurol.2012.242 23183883

[2] Fares MB Jagannath S Lashuel HA. Reverse engineering Lewy bodies: how far have we come and how far can we go? Nat Rev Neurosci. (2021) 22:111–31. 10.1038/s41583-020-00416-6 33432241

[3] Stephens AD Zacharopoulou M Kaminski Schierle GS. The cellular environment affects monomeric alpha-synuclein structure. Trends Biochem Sci. (2019) 44:453–66. 10.1016/j.tibs.2018.11.005 30527975

[4] Jankovic J Sherer T. The future of research in Parkinson disease. JAMA Neurol. (2014) 71:1351–2. 10.1001/jamaneurol.2014.1717 25178587

[5] Guo JL Lee VM. Cell-to-cell transmission of pathogenic proteins in neurodegenerative diseases. Nat Med. (2014) 20:130–8. 10.1038/nm.3457 24504409 PMC4011661

[6] Lauwers E Lalli G Brandner S Collinge J Compernolle V Duyckaerts C. Potential human transmission of amyloid beta pathology: surveillance and risks. Lancet Neurol. (2020) 19:872–8. 10.1016/S1474-4422(20)30238-6 32949547

[7] Kovacs GG Breydo L Green R Kis V Puska G Lorincz P. Intracellular processing of disease-associated alpha-synuclein in the human brain suggests prion-like cell-to-cell spread. Neurobiol Dis. (2014) 69:76–92. 10.1016/j.nbd.2014.05.020 24878508

[8] Fujiwara H Hasegawa M Dohmae N Kawashima A Masliah E Goldberg MS. alpha-Synuclein is phosphorylated in synucleinopathy lesions. Nat Cell Biol. (2002) 4:160–4. 10.1038/ncb748 11813001

[9] Anderson JP Walker DE Goldstein JM de Laat R Banducci K Caccavello RJ. Phosphorylation of Ser-129 is the dominant pathological modification of alpha-synuclein in familial and sporadic Lewy body disease. J Biol Chem. (2006) 281:29739–52. 10.1074/jbc.M600933200 16847063

[10] Chen L Feany MB. Alpha-synuclein phosphorylation controls neurotoxicity and inclusion formation in a Drosophila model of Parkinson disease. Nat Neurosci. (2005) 8:657–63. 10.1038/nn1443 15834418

[11] Paleologou KE Schmid AW Rospigliosi CC Kim HY Lamberto GR Fredenburg RA. Phosphorylation at Ser-129 but not the phosphomimics S129E/D inhibits the fibrillation of alpha-synuclein. J Biol Chem. (2008) 283:16895–905. 10.1074/jbc.M800747200 18343814 PMC2423264

[12] Sato H Arawaka S Hara S Fukushima S Koga K Koyama S. Authentically phosphorylated alpha-synuclein at Ser129 accelerates neurodegeneration in a rat model of familial Parkinson's disease. J Neurosci. (2011) 31:16884–94. 10.1523/JNEUROSCI.3967-11.2011 22090514 PMC6633319

[13] Colom-Cadena M Pegueroles J Herrmann AG Henstridge CM Munoz L Querol-Vilaseca M. Synaptic phosphorylated alpha-synuclein in dementia with Lewy bodies. Brain. (2017) 140:3204–14. 10.1093/brain/awx275 29177427 PMC5841145

[14] Ma MR Hu ZW Zhao YF Chen YX Li YM. Phosphorylation induces distinct alpha-synuclein strain formation. Sci Rep. (2016) 6:37130. 10.1038/srep37130 27853185 PMC5112567

[15] Karampetsou M Ardah MT Semitekolou M Polissidis A Samiotaki M Kalomoiri M. Phosphorylated exogenous alpha-synuclein fibrils exacerbate pathology and induce neuronal dysfunction in mice. Sci Rep. (2017) 7:16533. 10.1038/s41598-017-15813-8 29184069 PMC5705684

[16] Zhang S Liu YQ Jia C Lim YJ Feng G Xu E. Mechanistic basis for receptor-mediated pathological alpha-synuclein fibril cell-to-cell transmission in Parkinson's disease. Proc Natl Acad Sci USA. (2021) 118:e2011196118. 10.1073/pnas.2011196118 34172566 PMC8256039

[17] Tsukita K Sakamaki-Tsukita H Tanaka K Suenaga T Takahashi R. Value of in vivo alpha-synuclein deposits in Parkinson's disease: A systematic review and meta-analysis. Mov Disord. (2019) 34:1452–63. 10.1002/mds.27794 31322768

[18] Chahine LM Beach TG Brumm MC Adler CH Coffey CS Mosovsky S. In vivo distribution of alpha-synuclein in multiple tissues and biofluids in Parkinson disease. Neurology. (2020) 95:e1267–84. 10.1212/WNL.0000000000010404 32747521 PMC7538226

[19] Hansson O. Biomarkers for neurodegenerative diseases. Nat Med. (2021) 27:954–63. 10.1038/s41591-021-01382-x 34083813

[20] Braak H Del Tredici K Bratzke H Hamm-Clement J Sandmann-Keil D Rub U. Staging of the intracerebral inclusion body pathology associated with idiopathic Parkinson's disease (preclinical and clinical stages). J Neurol. (2002) 249:III/1–5. 10.1007/s00415-002-1301-4 12528692

[21] Braak H Del Tredici K Rub U de Vos RA Jansen Steur EN Braak E. Staging of brain pathology related to sporadic Parkinson's disease. Neurobiol Aging. (2003) 24:197–211. 10.1016/S0197-4580(02)00065-9 12498954

[22] Mao X Ou MT Karuppagounder SS Kam TI Yin X Xiong Y. Pathological alpha-synuclein transmission initiated by binding lymphocyte-activation gene 3. Science. (2016) 353:aah3374. 10.1126/science.aah3374 27708076 PMC5510615

[23] Luna E Decker SC Riddle DM Caputo A Zhang B Cole T. Differential alpha-synuclein expression contributes to selective vulnerability of hippocampal neuron subpopulations to fibril-induced toxicity. Acta Neuropathol. (2018) 135:855–75. 10.1007/s00401-018-1829-8 29502200 PMC5955788

[24] Pediaditakis I Kodella KR Manatakis DV Le CY Hinojosa CD Tien-Street W. Modeling alpha-synuclein pathology in a human brain-chip to assess blood-brain barrier disruption. Nat Commun. (2021) 12:5907. 10.1038/s41467-021-26066-5 34625559 PMC8501050

[25] Sacino AN Brooks M Thomas MA McKinney AB McGarvey NH Rutherford NJ. Amyloidogenic alpha-synuclein seeds do not invariably induce rapid, widespread pathology in mice. Acta Neuropathol. (2014) 127:645–65. 10.1007/s00401-014-1268-0 24659240 PMC4869357

[26] Rutherford NJ Brooks M Giasson BI. Novel antibodies to phosphorylated alpha-synuclein serine 129 and NFL serine 473 demonstrate the close molecular homology of these epitopes. Acta Neuropathol Commun. (2016) 4:80. 10.1186/s40478-016-0357-9 27503460 PMC4977832

[27] Delic V Chandra S Abdelmotilib H Maltbie T Wang S Kem D. Sensitivity and specificity of phospho-Ser129 alpha-synuclein monoclonal antibodies. J Comp Neurol. (2018) 526:1978–90. 10.1002/cne.24468 29888794 PMC6031478

[28] Mbefo MK Paleologou KE Boucharaba A Oueslati A Schell H Fournier M. Phosphorylation of synucleins by members of the Polo-like kinase family. J Biol Chem. (2010) 285:2807–22. 10.1074/jbc.M109.081950 19889641 PMC2807335

[29] Liu J Liu W Lu Y Tian H Duan C Lu L. Piperlongumine restores the balance of autophagy and apoptosis by increasing BCL2 phosphorylation in rotenone-induced Parkinson disease models. Autophagy. (2018) 14:845–61. 10.1080/15548627.2017.1390636 29433359 PMC6070010

[30] Volpicelli-Daley LA Luk KC Lee VM. Addition of exogenous alpha-synuclein preformed fibrils to primary neuronal cultures to seed recruitment of endogenous alpha-synuclein to Lewy body and Lewy neurite-like aggregates. Nat Protoc. (2014) 9:2135–46. 10.1038/nprot.2014.143 25122523 PMC4372899

[31] Luk KC Kehm V Carroll J Zhang B O'Brien P Trojanowski JQ. Pathological alpha-synuclein transmission initiates Parkinson-like neurodegeneration in nontransgenic mice. Science. (2012) 338:949–53. 10.1126/science.1227157 23161999 PMC3552321

[32] Mahul-Mellier AL Burtscher J Maharjan N Weerens L Croisier M Kuttler F. The process of Lewy body formation, rather than simply alpha-synuclein fibrillization, is one of the major drivers of neurodegeneration. Proc Natl Acad Sci USA. (2020) 117:4971–82. 10.1073/pnas.1913904117 32075919 PMC7060668

[33] Trier NH Hansen PR Houen G. Production and characterization of peptide antibodies. Methods. (2012) 56:136–44. 10.1016/j.ymeth.2011.12.001 22178691

[34] Stephens AD Zacharopoulou M Moons R Fusco G Seetaloo N Chiki A. Extent of N-terminus exposure of monomeric alpha-synuclein determines its aggregation propensity. Nat Commun. (2020) 11:2820. 10.1038/s41467-020-16564-3 32499486 PMC7272411

[35] Tian H Lu Y Liu J Liu W Lu L Duan C. Leucine Carboxyl Methyltransferase Downregulation and Protein Phosphatase Methylesterase Upregulation Contribute Toward the Inhibition of Protein Phosphatase 2A by alpha-Synuclein. Front Aging Neurosci. (2018) 10:173. 10.3389/fnagi.2018.00173 29950985 PMC6008559

[36] Aspholm EE Matecko-Burmann I Burmann BM. Keeping alpha-Synuclein at Bay: a more active role of molecular chaperones in preventing mitochondrial interactions and transition to pathological states? Life. (2020) 10:289. 10.3390/life10110289 33227899 PMC7699229

[37] Burmann BM Gerez JA Matecko-Burmann I Campioni S Kumari P Ghosh D. Regulation of alpha-synuclein by chaperones in mammalian cells. Nature. (2020) 577:127–32. 10.1038/s41586-019-1808-9 31802003 PMC6930850

[38] Haque ME Akther M Azam S Kim IS Lin Y Lee YH. Targeting alpha-synuclein aggregation and its role in mitochondrial dysfunction in Parkinson's disease. Br J Pharmacol. (2022) 179:23–45. 10.1111/bph.15684 34528272

[39] Nakamura K Nemani VM Azarbal F Skibinski G Levy JM Egami K. Direct membrane association drives mitochondrial fission by the Parkinson disease-associated protein alpha-synuclein. J Biol Chem. (2011) 286:20710–26. 10.1074/jbc.M110.213538 21489994 PMC3121472

[40] Wang X Becker K Levine N Zhang M Lieberman AP Moore DJ. Pathogenic alpha-synuclein aggregates preferentially bind to mitochondria and affect cellular respiration. Acta Neuropathol Commun. (2019) 7:41. 10.1186/s40478-019-0696-4 30871620 PMC6419482

[41] Malpartida AB Williamson M Narendra DP Wade-Martins R Ryan BJ. Mitochondrial Dysfunction and Mitophagy in Parkinson's Disease: From Mechanism to Therapy. Trends Biochem Sci. (2021) 46:329–43. 10.1016/j.tibs.2020.11.007 33323315

[42] McFarland MA Ellis CE Markey SP Nussbaum RL. Proteomics analysis identifies phosphorylation-dependent alpha-synuclein protein interactions. Mol Cell Proteomics. (2008) 7:2123–37. 10.1074/mcp.M800116-MCP200 18614564 PMC2577212

[43] Grassi D Diaz-Perez N Volpicelli-Daley LA Lasmezas CI. Palpha-syn^*^ mitotoxicity is linked to MAPK activation and involves tau phosphorylation and aggregation at the mitochondria. Neurobiol Dis. (2019) 124:248–62. 10.1016/j.nbd.2018.11.015 30472299

[44] Huang Z Xu Z Wu Y Zhou Y. Determining nuclear localization of alpha-synuclein in mouse brains. Neuroscience. (2011) 199:318–32. 10.1016/j.neuroscience.2011.10.016 22033456 PMC3237852

[45] Davidi D Schechter M Elhadi SA Matatov A Nathanson L Sharon R. alpha-Synuclein Translocates to the Nucleus to Activate Retinoic-Acid-Dependent Gene Transcription. iScience. (2020) 23:100910. 10.1016/j.isci.2020.100910 32120069 PMC7052517

[46] Pinho R Paiva I Jercic KG Fonseca-Ornelas L Gerhardt E Fahlbusch C. Nuclear localization and phosphorylation modulate pathological effects of alpha-synuclein. Hum Mol Genet. (2019) 28:31–50. 10.1093/hmg/ddy326 30219847

[47] Dent SE King DP Osterberg VR Adams EK Mackiewicz MR Weissman TA. Phosphorylation of the aggregate-forming protein alpha-synuclein on serine-129 inhibits its DNA-bending properties. J Biol Chem. (2021) 101552. 10.1016/j.jbc.2021.101552 34973339 PMC8800120

[48] Shaltouki A Hsieh CH Kim MJ Wang X. Alpha-synuclein delays mitophagy and targeting Miro rescues neuron loss in Parkinson's models. Acta Neuropathol. (2018) 136:607–20. 10.1007/s00401-018-1873-4 29923074 PMC6123262

