# Supplementary figures and images for: Characterization of a Novel Monoclonal Antibody for Serine-129 Phosphorylated α-Synuclein: A Potential Application for Clinical and Basic Research
Source: Front Neurol. 2022 Feb 17;13:821792. doi: 10.3389/fneur.2022.821792 (PMC8893957; doi:10.3389/fneur.2022.821792)

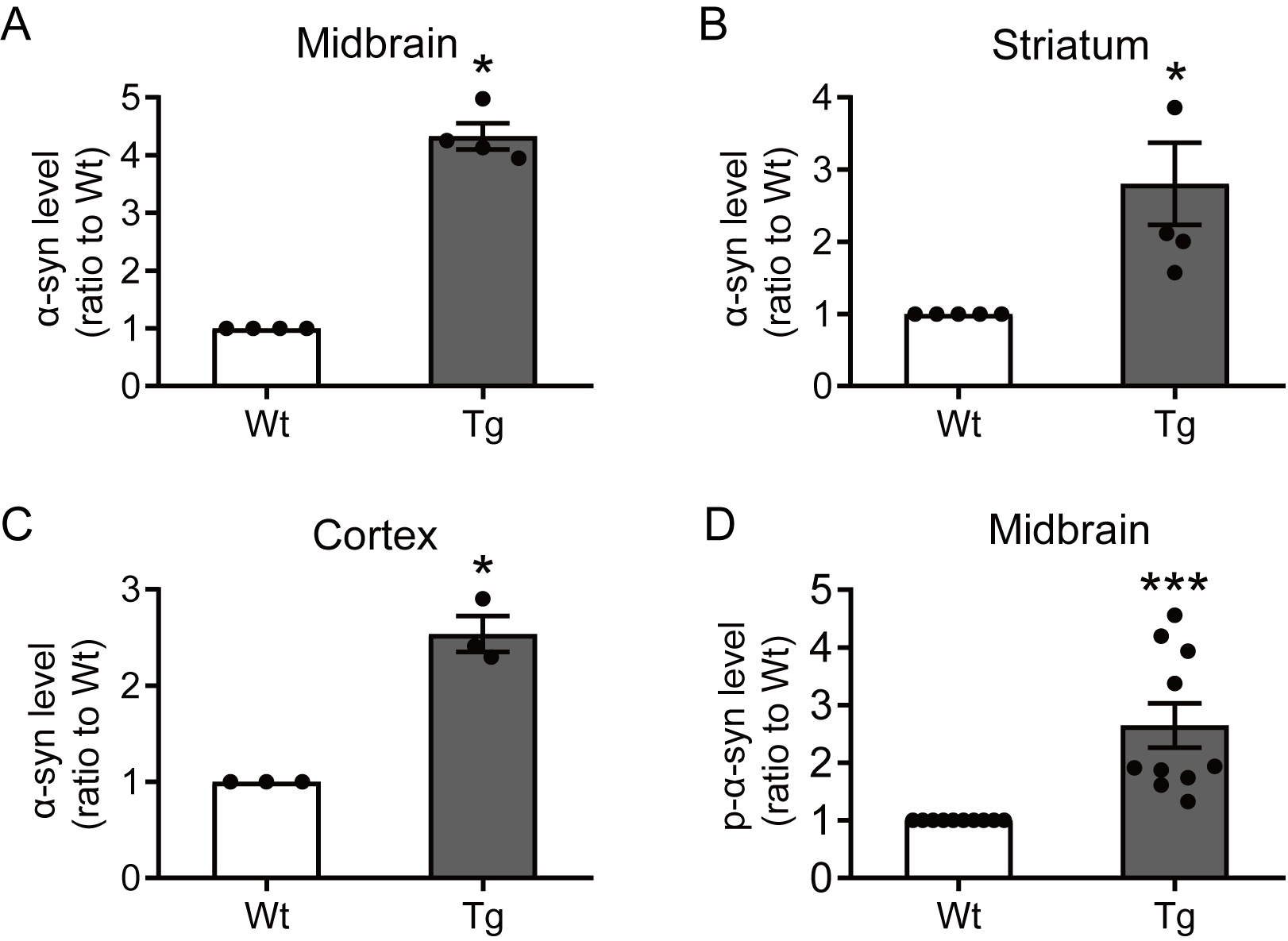

Supplement: Supplementary Figure S1 — Quantification of band intensities of α-syn and p-α-syn in the brains of Wt/Tg mice. (A–C) Quantification of the band intensities of α-syn levels in the midbrain (A)/striatum (B)/cortex (C) from wild-type brood (Wt) and Thy1-SNCA transgenic (Tg) mice (Figures 4D–F). (D) Quantification of the band intensities (17 kD) of p-α-syn levels in the midbrains of Wt and Tg mice (Figures 4G–I). The results are expressed as the mean ± standard error of mean (SEM) (unpaired t-test, n = 3–10). *P < 0.05, ***P < 0.001 vs. Wt. Mice age: 13-months-old. [file Image_1.TIF]

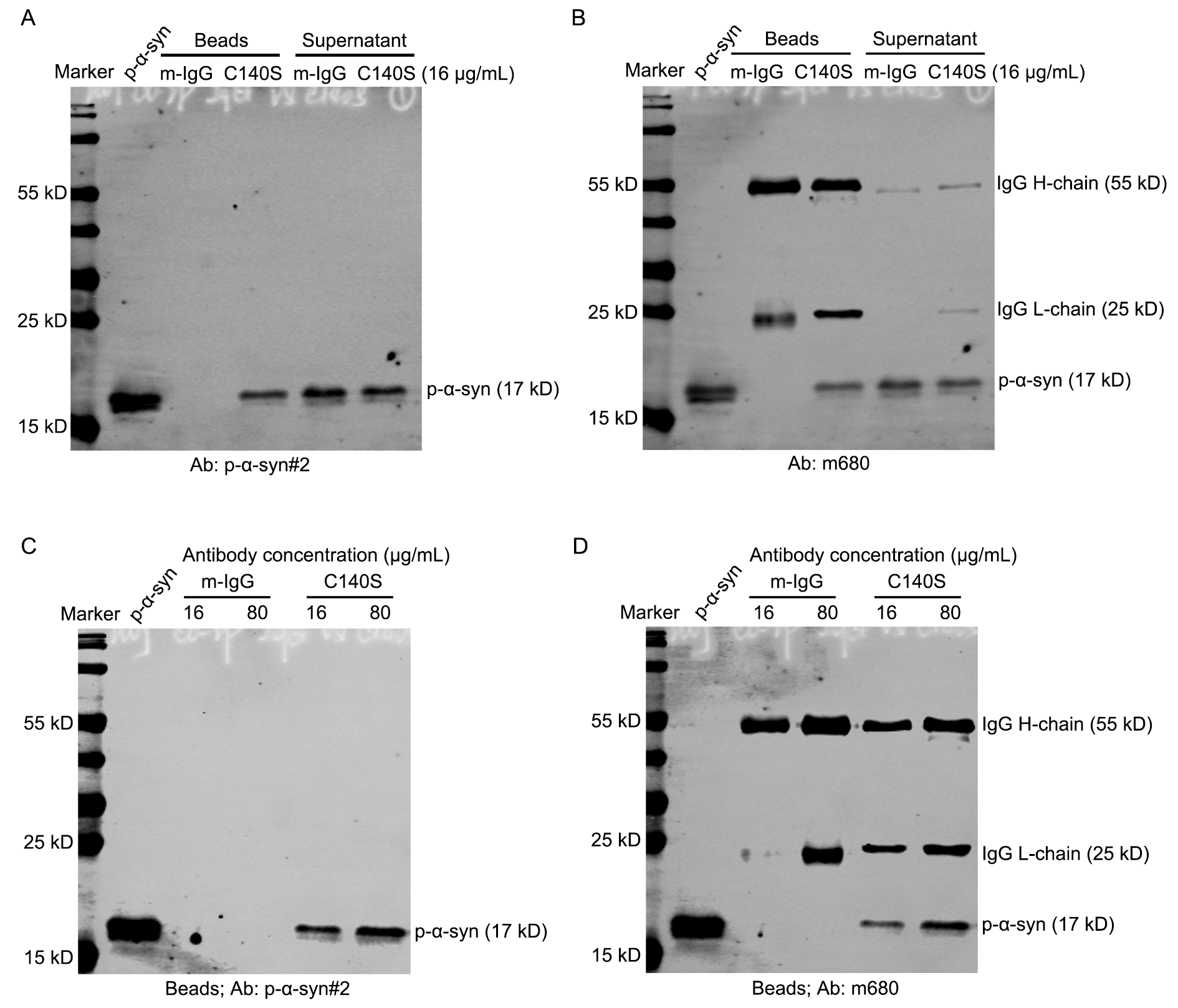

Supplement: Supplementary Figure S2 — The specificity and affinity of C140S were confirmed by antibody absorption experiment. (A,B) A total of 5 μg h of p-α-syn was incubated with control mouse IgG (m-IgG) and C140S antibody (16 μg/mL). The protein G beads were added to conjugate with the antigen-antibody complex. The beads-antigen-antibody complex and supernatant were separated and examined using western bolts, shown in (A,B). The p-α-syn was detected with p-α-syn#2 antibody (Abcam, Cambridge, UK), m-IgG was detected with fluorophore-conjugated secondary antibody mouse 680 (m680). (C,D) A total of 5 μg h of p-α-syn was incubated with m-IgG and C140S antibody (16, 80 μg/mL). The protein G beads were added to conjugate with the antigen-antibody complex. The beads-antigen-antibody complex was separated and examined using western bolts, shown in (C,D). The p-α-syn was detected with p-α-syn#2 antibody (Abcam, Cambridge, UK), m-IgG was detected with m680. The p-α-syn protein was added as a positive control. Ab, Antibody. [file Image_2.TIF]
